# Supplementary material for: Plasma membrane H+-ATPase overexpression increases rice yield via simultaneous enhancement of nutrient uptake and photosynthesis
Source: Nat Commun. 2021 Feb 2;12:735. doi: 10.1038/s41467-021-20964-4 (PMC7854686; doi:10.1038/s41467-021-20964-4)
Supplement: Supplementary file 1 — Supplementary Information [file 41467_2021_20964_MOESM1_ESM.pdf]

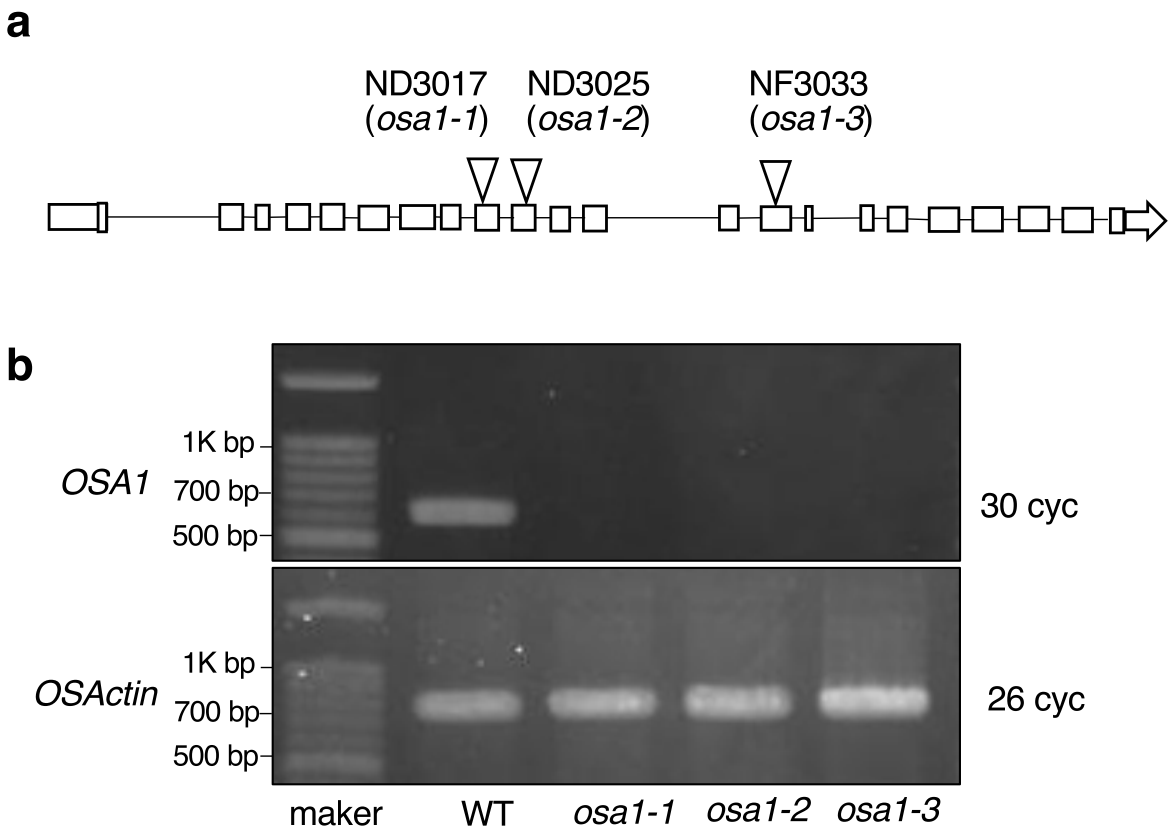

Supplementary Figure 1 TOS17 insertion site and RT-PCR of *OSA1* in *osa1* mutants. a Schematic diagram of the genome structure of *OSA1*. Lines represent UTRs and introns. Exons are represented by white boxes. Positions of the *TOS17* insertion are indicated by small triangles. **b** *OSA1* expression in the roots of WT and *osa1* mutants by RT-PCR. Experiments were repeated three occasions with similar results.

Supplementary Figure 2

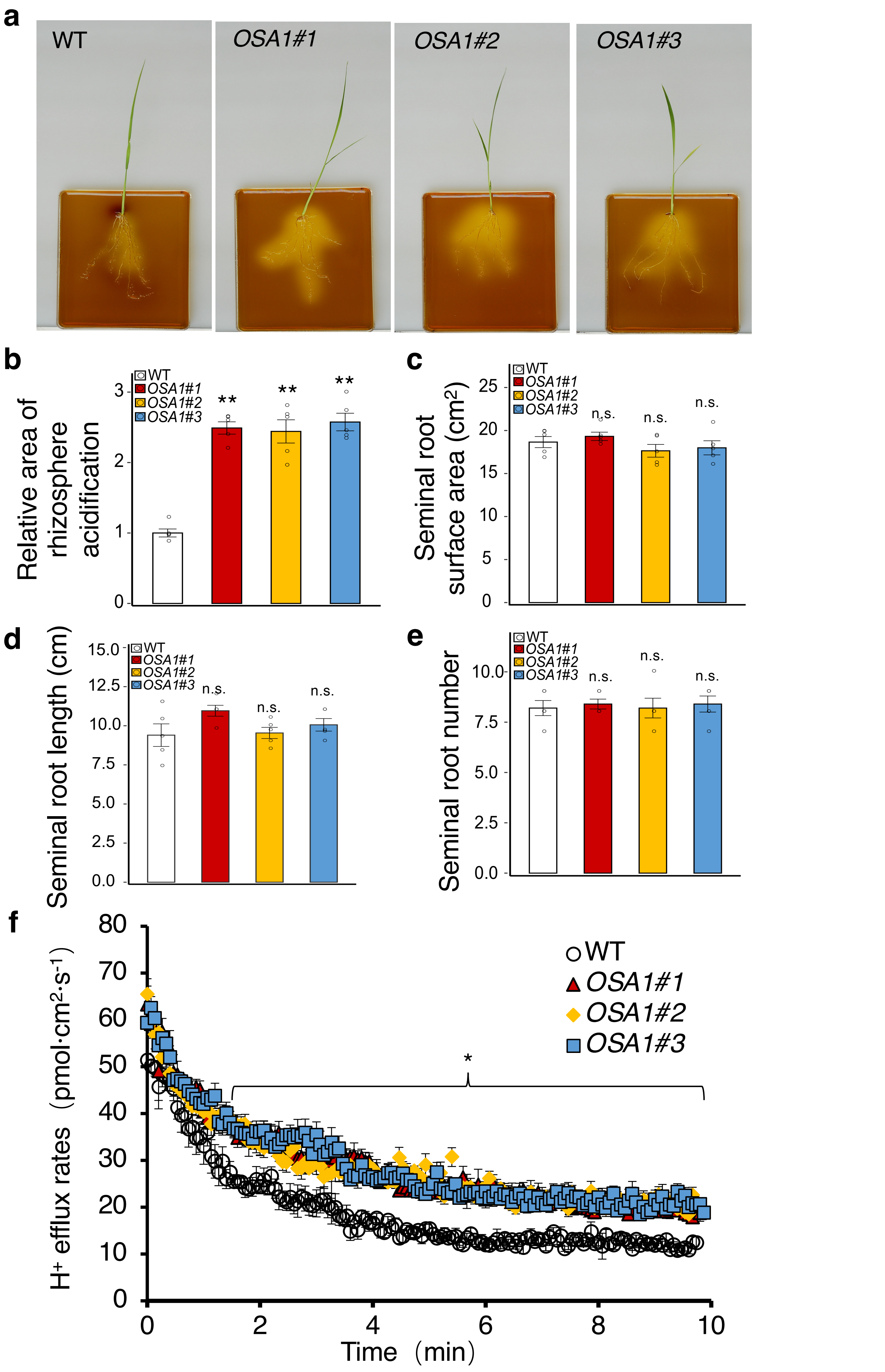

Supplementary Figure 2 Rhizosphere acidification and H^+ efflux properties of WT and *OSA1*-oxs rice. **a** Monitoring of rhizosphere acidification around WT and *OSA1*-oxs rice roots. Plants were grown in nutrient solution for 7 days. After washing with deionized water, roots were carefully spread onto solid medium containing 0.02% (w/v) bromocresol purple and 0.7% (w/v) agar adjusted to pH 5.6. After incubation for 12 hr in the dark, the plates were photographed. **b** Relative area of rhizosphere acidification (yellow area) on the surface of agar plates. **c-e** Surface area (**c**), length(**d**) and number (**e**) of seminal roots in WT and *OSA1*-oxs. Small circles represent the data points of individual experiments were performed. Values are mean ± SEs (n = 5). **f** Quantification of H^+ efflux rates from WT and *OSA1*-oxs roots. Intact roots (3-5 cm in length from the root tips) were equilibrated in the measuring solution for 10 min. Values are mean ± SEs (n = 6). Differences were evaluated using the two-tailed Student's *t*-test ($^*P < 0.05$; $^{**}P < 0.01$, n.s., not significant). The exact p values are provided in the Supplementary Data 5.

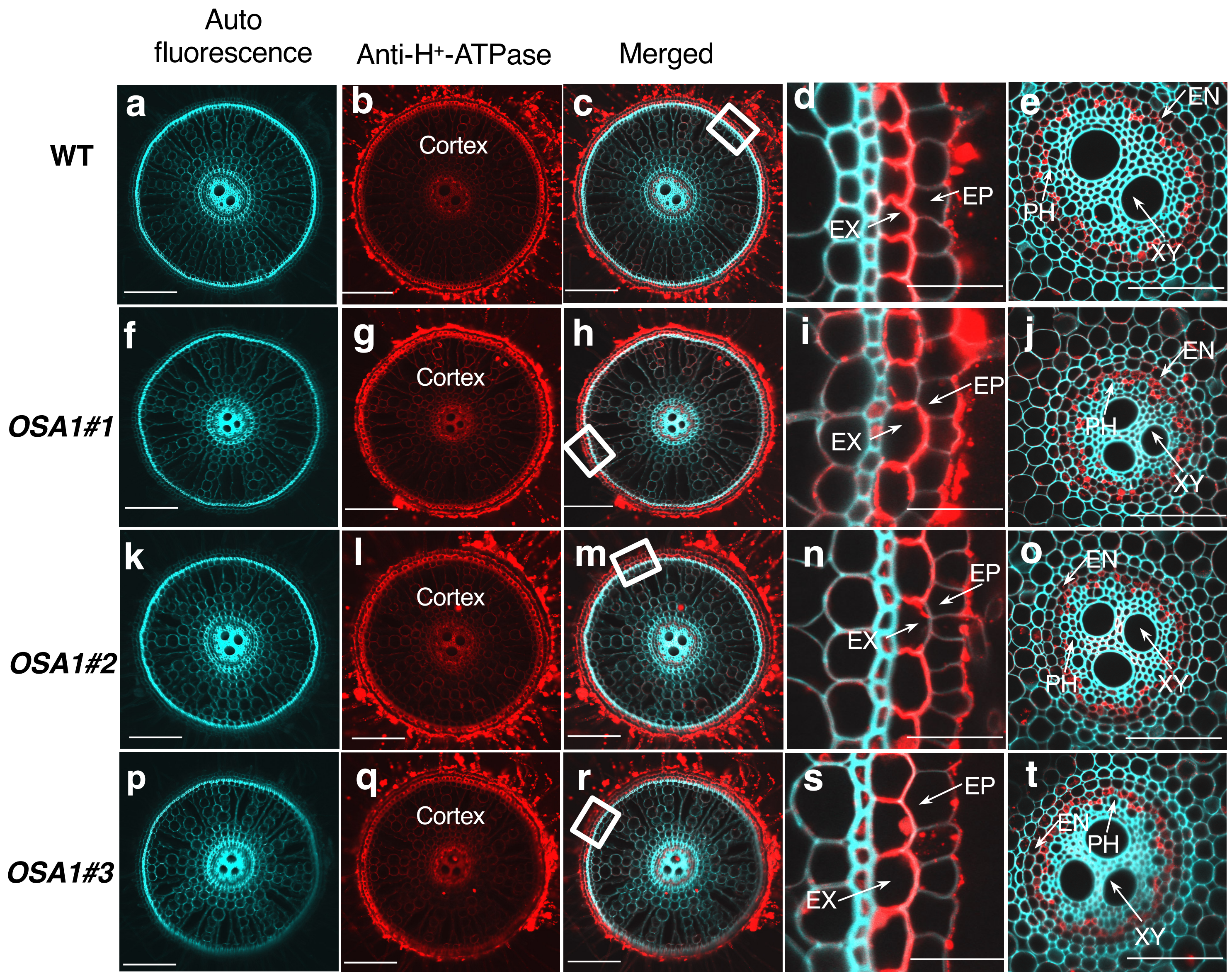

Supplementary Figure 3 Localization of PM H^{+} ATPase in rice roots. Immunohistochemical staining of 3-weeks-old seedlings in WT and *OSA1*-oxs, with polyclonal antibodies recognizing rice PM H^{+}-ATPase (anti-H^{+}-ATPase) was performed in rice root. Red color shows the signal of anti-H^{+}-ATPase (**b**, **j**), blue color shows autofluorescence of cell wall stained by DAPI(**a**, **f**), and the merged image (**c**-**e** and **h**-**j**). EP, epidermis; EX, exodermis; EN, endodermis; PH, phloem; XY, xylem. Bars = 150 μm (**a**-**c**, **f**-**h**, **k**-**m** and **p**-**r**) ,20 μm (**d**, **i**, **n** and **s**) and 60 μm (**e**, **j**, **o** and **t**). Experiments were repeated three occasions (**a**-**t**) with similar results.

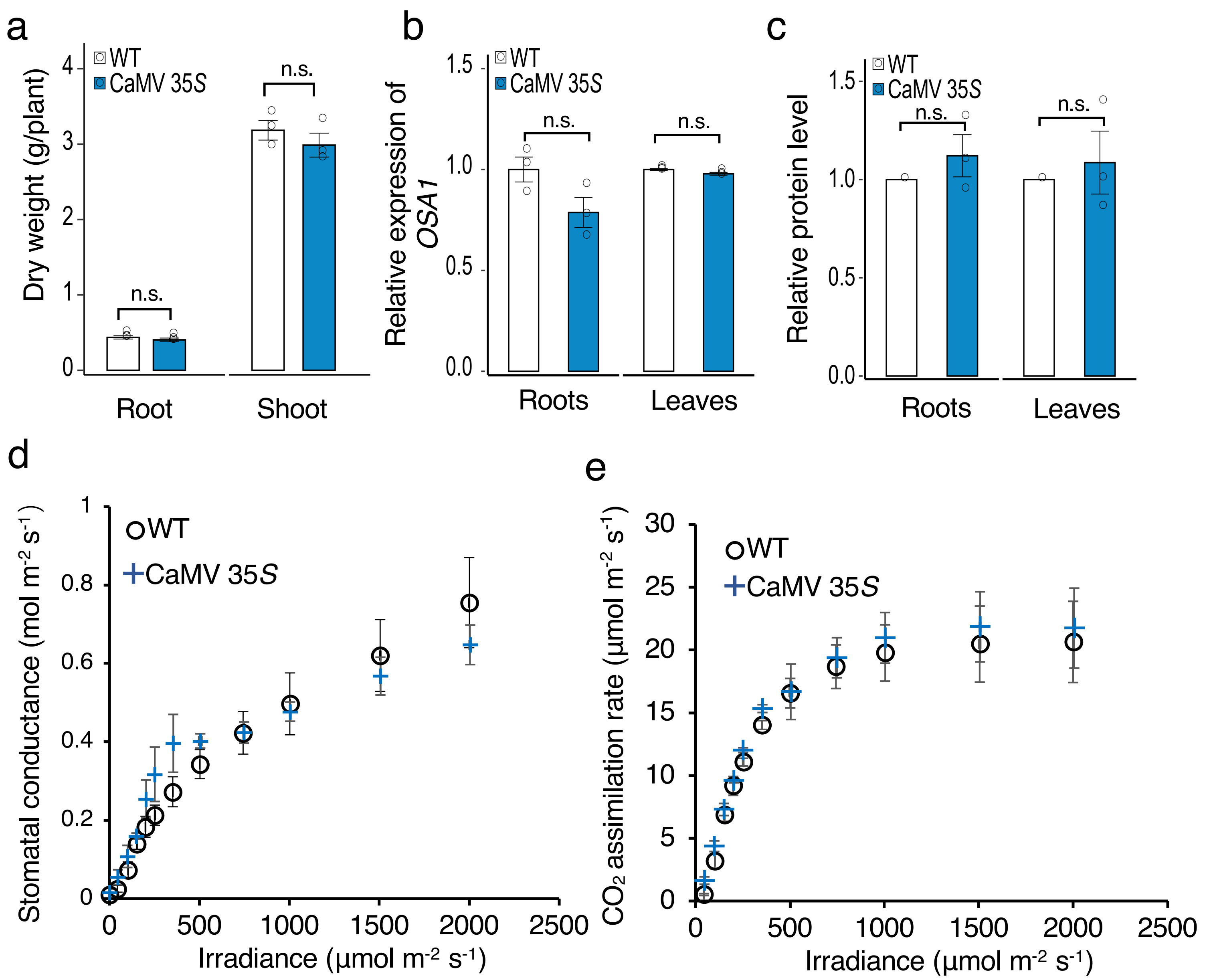

Supplementary Figure 4 Plant growth and gas-exchange properties in CaMV 35*S* empty vector transformed rice. **a** Dry weights of root and shoot in WT and CaMV 35*S* empty vector transformed plants. **b** Relative expression level of *OSA1* in the roots and leaves of WT and CaMV 35*S* empty vector transformed plants. **c** Relative PM H^+-ATPase protein levels in the roots and leaves WT and CaMV 35*S* empty vector transformed plants. **d, e** Stomatal conductance (d) and CO_2 assimilation rate (e) in response to light in WT and CaMV 35*S* empty vector transformed plants. Small circles in (**a-c**) represent the data points for individual experiments and three biological replicates were performed. Values in (**a**–**c**) are presented as the means ± SEs (n = 3) and those in (**d**, **e**) are the means ± standard deviations (n = 3). Differences in (a–c) were evaluated using the two-tailed Student's t-test (*$P < 0.05$; ** $P < 0.01$).

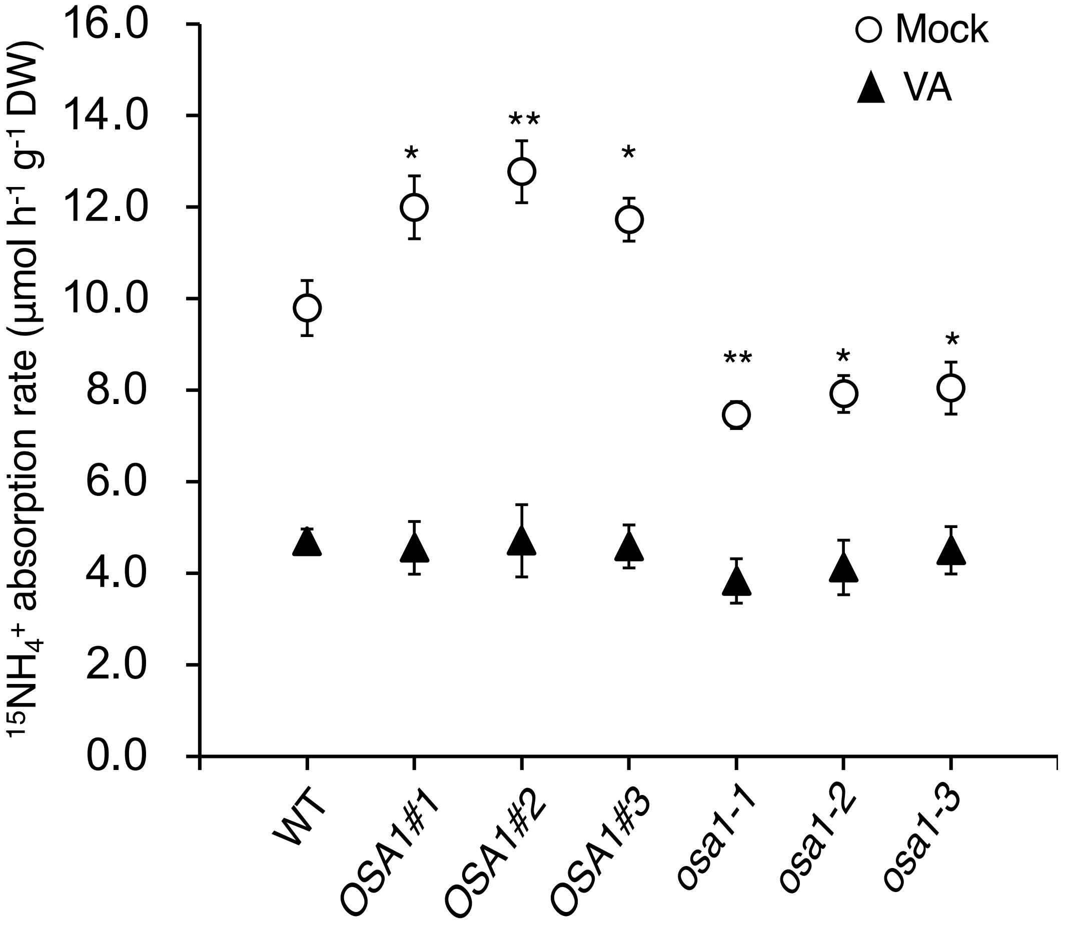

Supplementary Figure 5 ^{15}N absorption rate by WT and *OSA1*-oxs, and *osa1* mutants. Rice plants were grown hydroponically in a greenhouse for 4 weeks. To determine $^{15}NH_4^+$ absorption rates, seedlings were incubated in 2 mM $^{15}NH_4^+$ solution for 30 min under darkness with the treatment of 350 µM vanadate (VA) for rice roots. Values are the mean ± SEs ($n = 3$). Differences were evaluated using the two-tailed Student's *t*-test (*$P < 0.05$; ** $P < 0.01$). The exact p values are provided in the Supplementary Data 5.

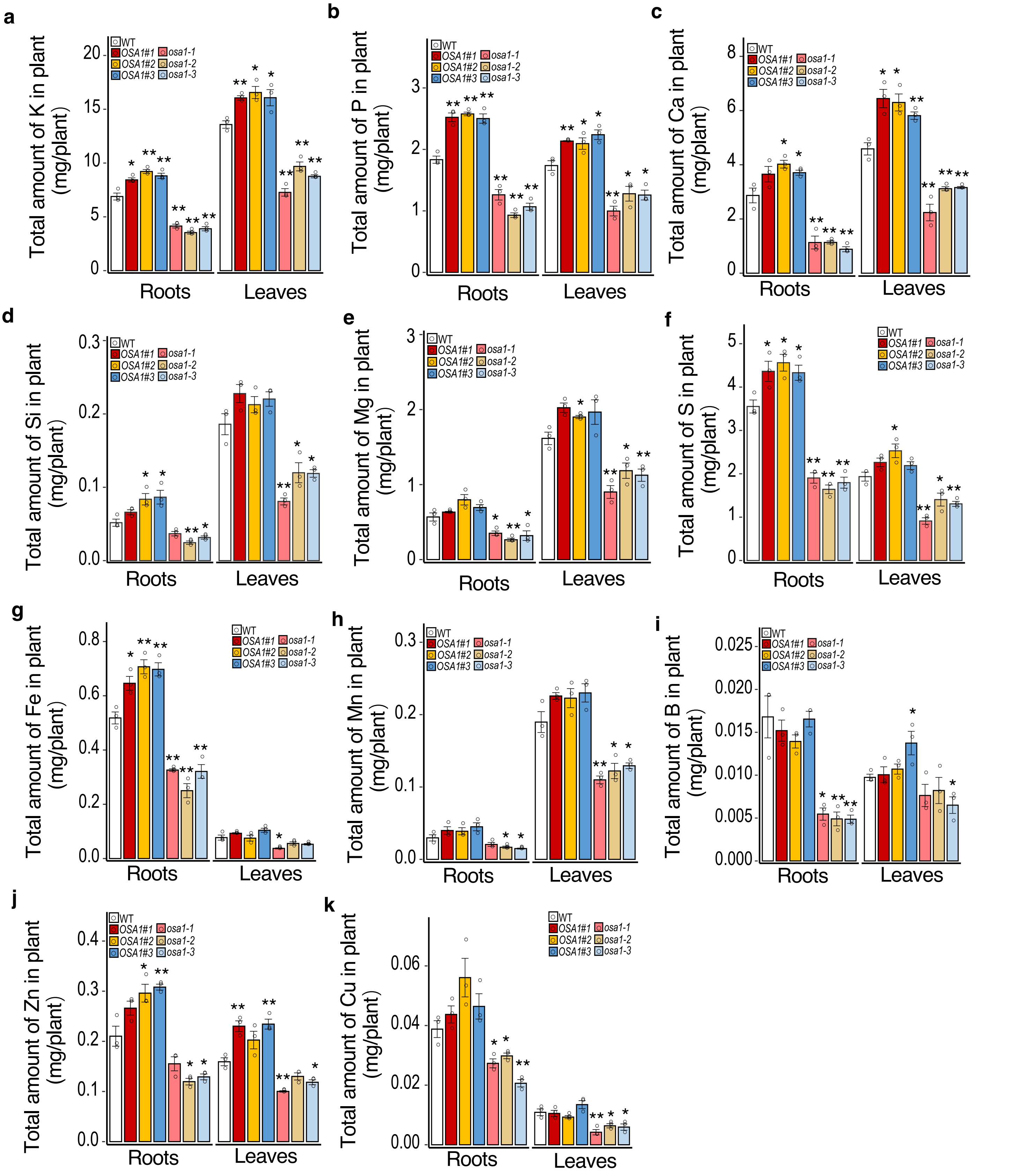

Supplementary Figure 6 *OSA1* overexpression promotes nutrition elements uptake in rice. a-k Total amount of K (**a**) , P (**b**) ,Ca (**c**) , Si (**d**) ,Mg (**e**) , S (**f**) ,Fe (**j**) , Mn (**h**) ,B (**i**) , Zn (**j**) and Cu (**k**) in WT and *OSA1*-ox and *osa1* mutant plants. Plants were grown hydroponically in a greenhouse for 3 weeks. Small circles in (**a-k**) represent the data points for individual experiments and three biological replicates were performed. Values in (**a**–**k**) are presented as the means ± SEs (n = 3). Differences were evaluated using the two-tailed Student's *t*-test (*$P < 0.05$; ** $P < 0.01$). The exact p values are provided in the Supplementary Data 5.

Supplementary Figure 7

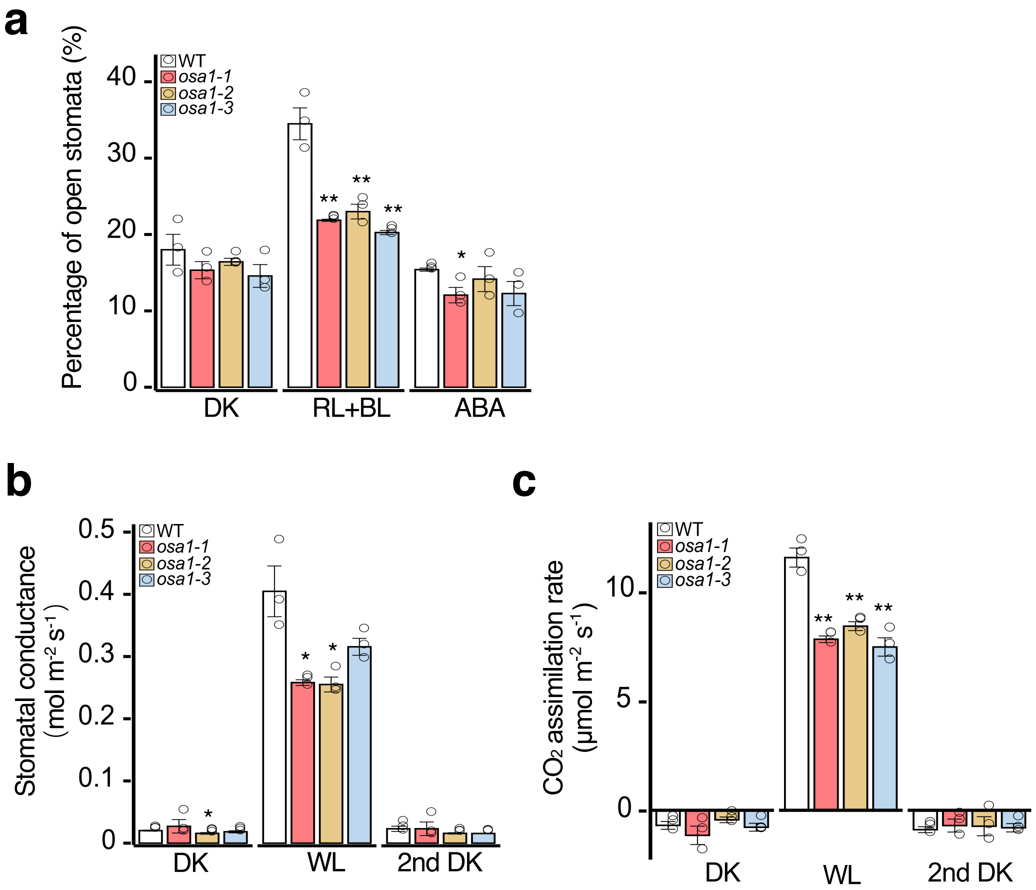

Supplementary Figure 7 Gas-exchange properties of *osa1* mutants. a Percentage of open stomata observed after 3 h of DK, RL+BL, or RL+BL in the presence of 20 µM ABA in WT. Differences were assessed using the Student's *t*-test. Error bars represent the SE (n = 3; at least 100 stomata observed under each condition). **b**, **c** Stomatal conductance (**b**) and CO_2 assimilation rate (**c**) in the DK (30 min), WL (2 h), and a 2nd DK treatment (30 min) in WT and *osa1* mutants, where light intensity was 1,000 µmol m^{-2} s^{-1}. Small circles in (a) to (c) represent the data points for individual experiments and three biological replicates were performed.Values in (**a**–**c**) are presented as the means ± SEs (n = 3). Differences in (**a**–**c**) were evaluated using the two-tailed Student's *t*-test (**P* < 0.05; ** *P*< 0.01). The exact p values are provided in the Supplementary Data 5.

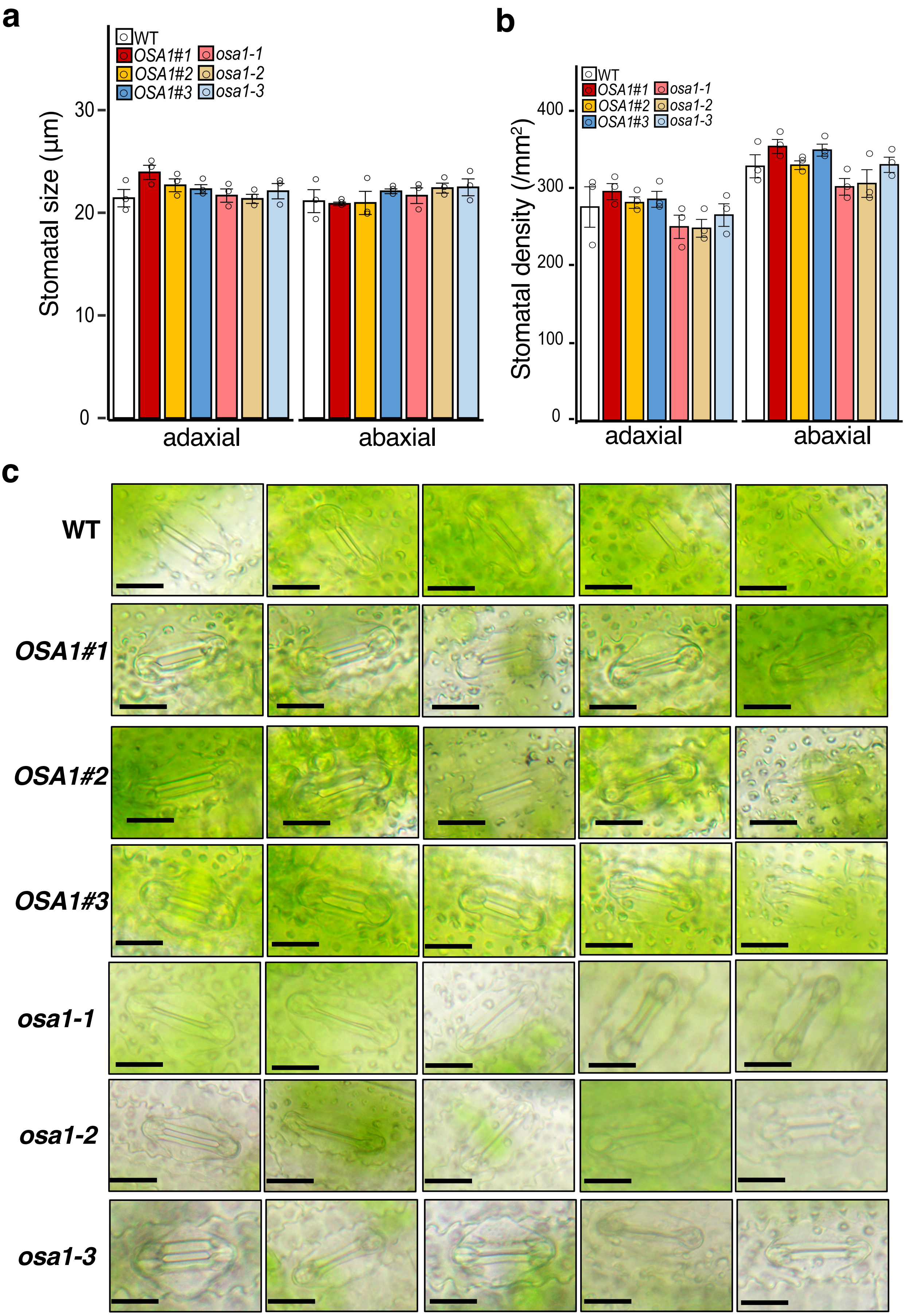
a
WT
OSA1#1
OSA1#2
OSA1#3
osa1-1
osa1-2
osa1-3
Stomatal size (µm)
0
10
20
30
adaxial
abaxial
b
Stomatal density (/mm²)
0
100
200
300
400
adaxial
abaxial
c
WT
OSA1#1
OSA1#2
OSA1#3
osa1-1
osa1-2
osa1-3

Bar = 8 µm

Supplementary Figure 8 Stomatal density, size, and shape in WT, *OSA1*-oxs and *osa1* mutant plants. **a** Stomatal size in WT, *OSA1*-oxs, and *osa1* mutants (in each experiment, 60 stomata were examined). **b** Stomatal density in WT, *OSA1*-oxs, and *osa1* mutants (in each experiment, 5 microphotographs were examined). **c** Randomly selected stomata observed after 3 h of red light and blue light (RL+BL) in WT, *OSA1*-oxs, and *osa1* mutants. Experiments were repeated three occasions with similar results. Small circles in (**a**) and (**b**) represent the data points for individual experiments and three biological replicates were performed. Values in (**a**, **b**) are presented as the means ± SEs (n = 3). Differences in (**a**, **b**) were evaluated using the two-tailed Student's *t*-test, and no significant difference was found.

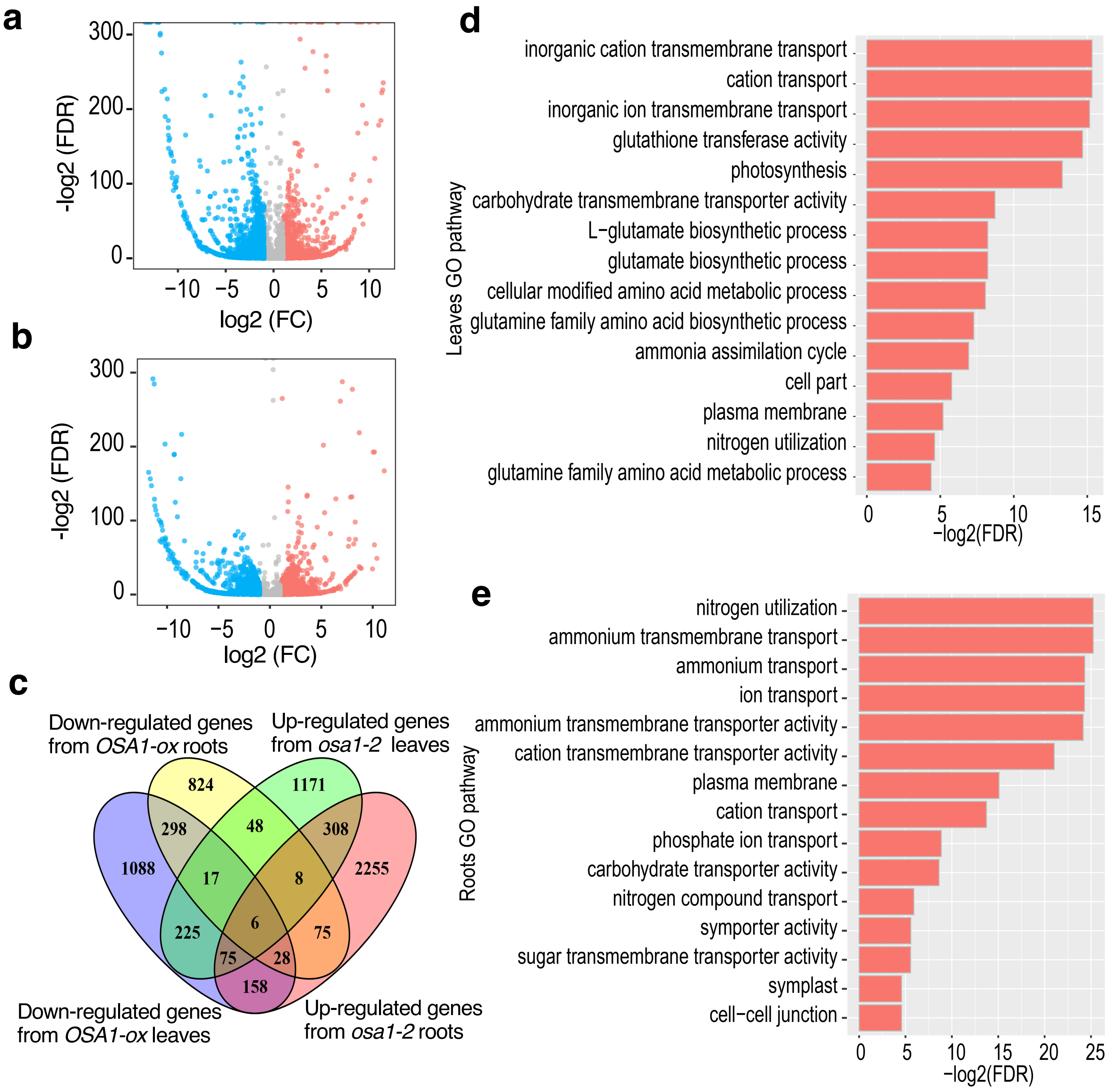

Supplementary Figure 9 The DEGs and GO enrichment caused by modification of *OSA1*. Plants were grown hydroponically in a greenhouse for 4 weeks. **a** DEGs of *OSA1*-ox plants in leaves. **b** DEGs of *OSA1*-ox plants in roots. Small pink circles in (a-b) represent the up-regulation of individual gene, blue circles represent the down-regulation gene, grey circles represent the not significantly regulated gene. **c** Venn diagram representing the overlaps of the down-regulated genes in *OSA1-ox* and the up-regulated genes in *osa1-2* mutant (*osa1*) from roots and leaves (*FDR <0.05*). **d-e** The significant GO analysis of genes in leaves (d) and roots (e), which were up regulated by *OSA1* overexpression, and down regulated by mutation of *OSA1* (*FDR*< 0.05).

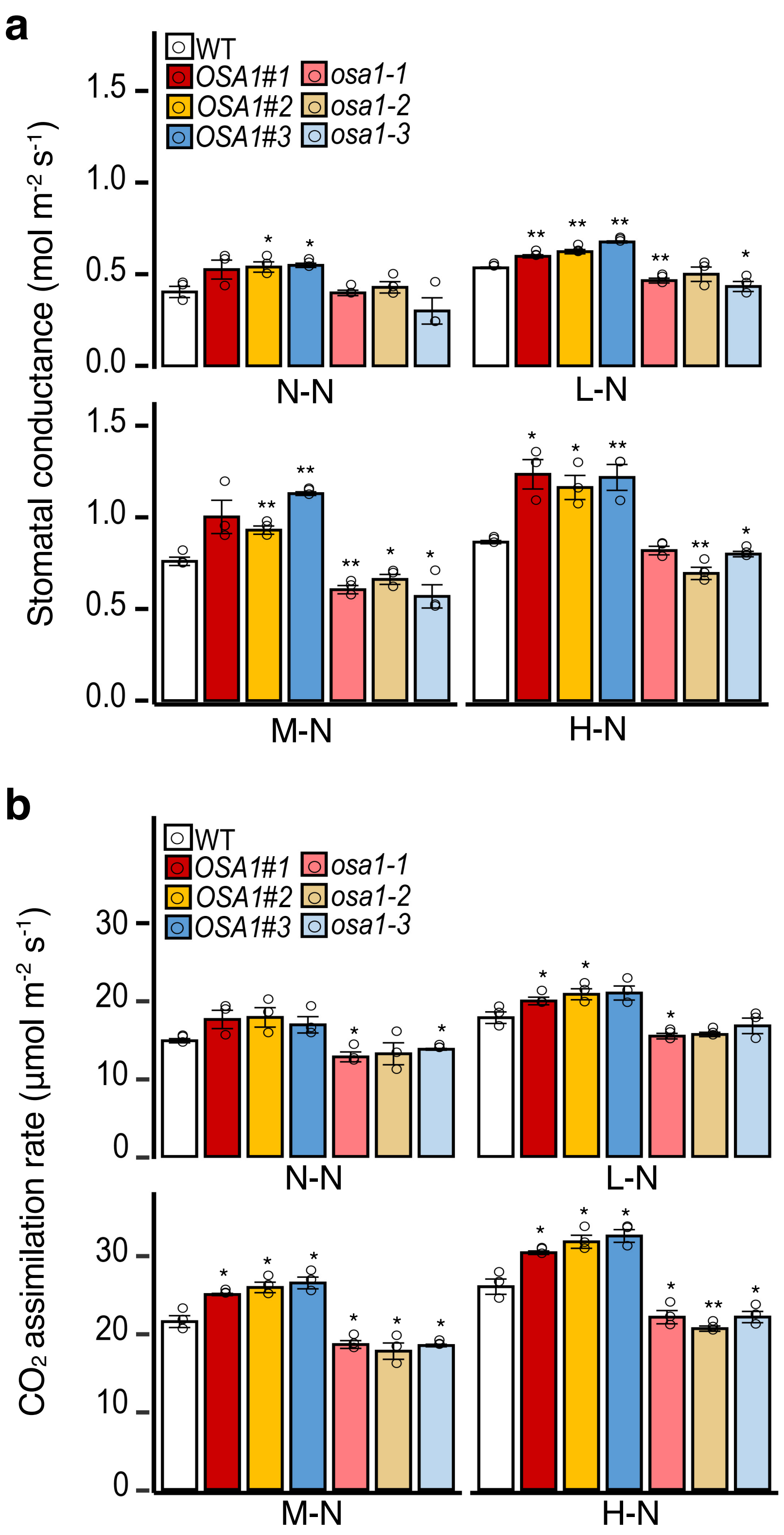

Supplementary Figure 10 Gas-exchange properties of WT, *OSA1*-oxs, and mutant plants in the field. Stomatal conductance (**a**) and net photosynthetic rate (**b**) in WT, *OSA1*-oxs, and *osa1* mutants. Plants were grown in the summer of 2017 at Nanjing-N under N-N (0 kg N/ha), L-N (100 kg N/ha), M-N (200 kg N/ha), or H-N (300 kg N/ha) fertilisation. Measurements were conducted at ambient [CO_2] (~400 μL L^{-1}) at the flowering stage. Leaf temperature and relative humidity in the leaf chamber were maintained at 28°C and 40–50% (Pa/Pa), respectively. Small circles in (**a**) and (**b**) represent the data points for individual experiments and three replicates were performed. Values in (**a**, **b**) are presented as the means ± SEs (n = 3). Differences were evaluated using the two-tailed Student's *t*-test (**P* < 0.05; ** *P* < 0.01). The exact p values are provided in the Supplementary Data 5.

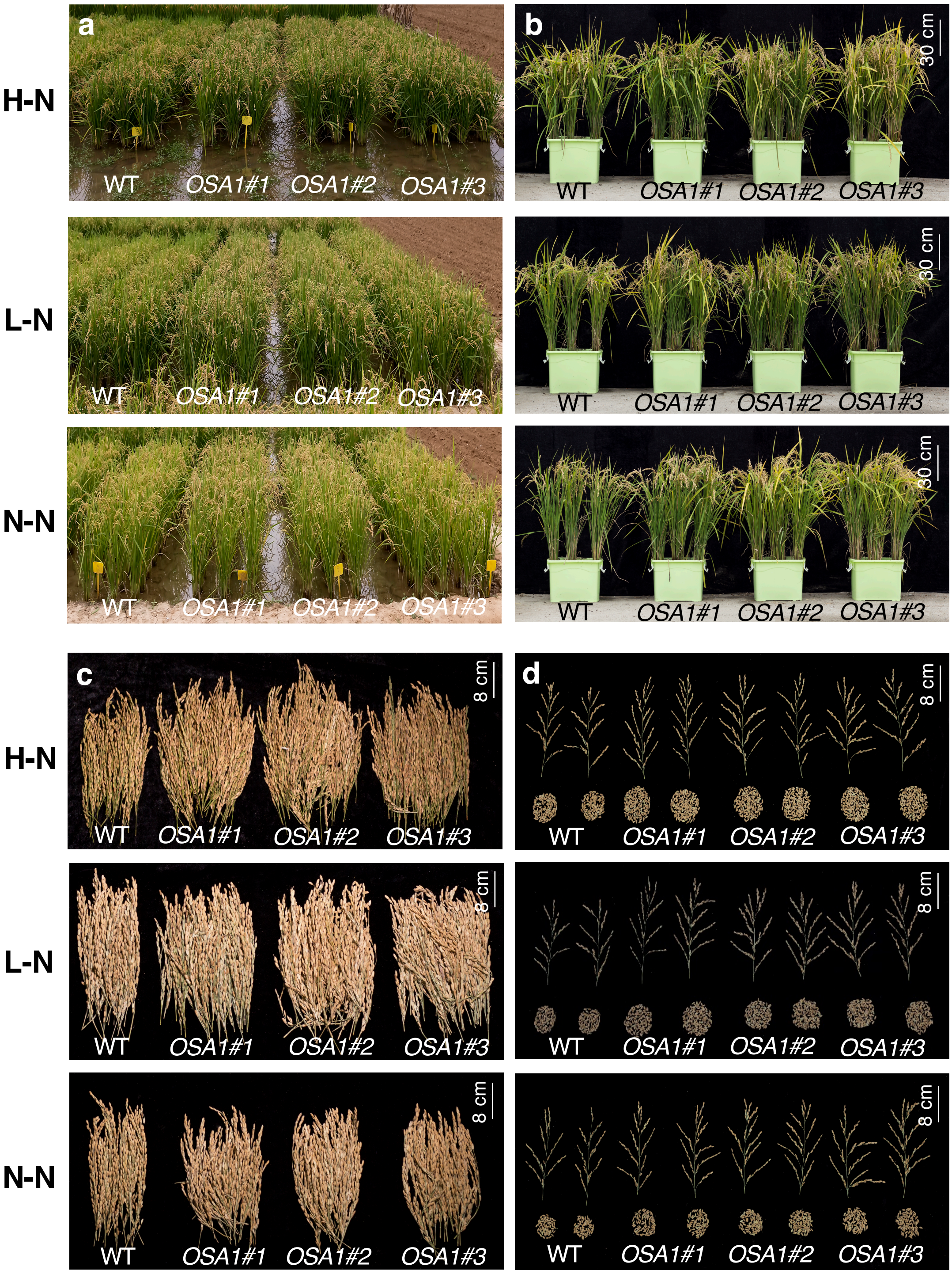

Supplementary Figure 11 Overexpression of *OSA1* increases grain yield in the field. a-d Photographs of 100-day-old WT and *OSA1*-oxs plants under H-N (300 kg N/ha), L-N (100 kg N/ha), or N-N (0 kg N/ha) fertilisation in the field (**a**) and in pots (**b**) in the summer of 2017 at Nanjing-N. **c**, **d** panicles (**c**) and spikelets (**d**) under different N fertilisation levels.

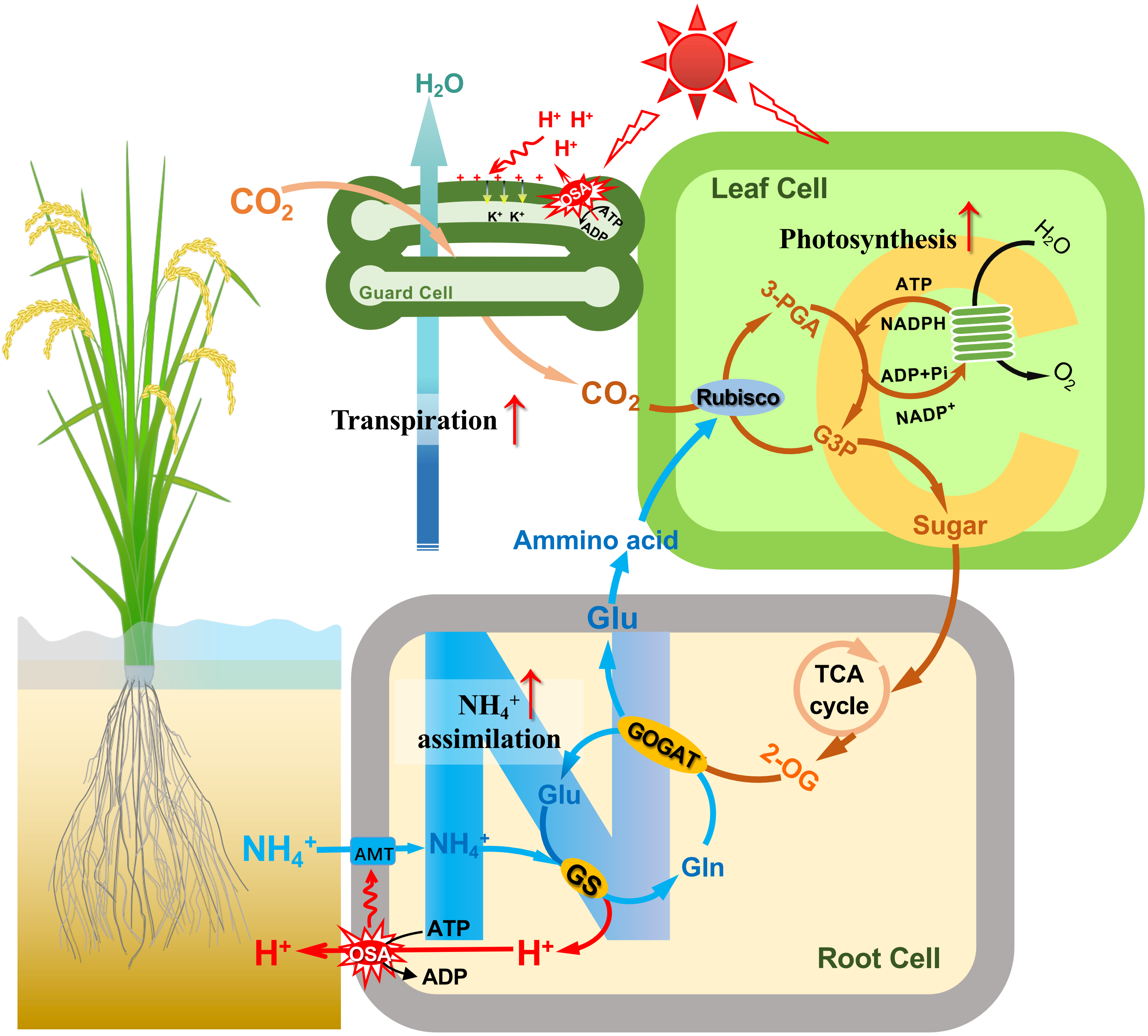

Supplementary Figure 12 Schematic diagram of the model for the effect of overexpression of *OSA1* on the interaction between C and N uptake and metabolism in paddy rice. Overexpression of *OSA1* in roots facilitates NH_4^+ uptake and also pumps excessive H^+ outside the root cells to guarantee the assimilation of NH_4^+ in cytoplasm. As the result, efficient utilization of NH_4^+ in rice roots provide amino acids and protein for the plant growth and photosynthesis. On the other side, overexpression of *OSA1* in guard cells polarized the membrane potential and trigger the K^+ influx for the opening of stomatal under light, which enhanced the uptake of CO_2 and improve the photosynthesis, which can provide carbon skeleton (2-OG) for the assimilation of NH_4^+ in roots. In addition, the enhanced transpiration by stomatal opening also improves the nutrients uptake due to the accelerated water transport in plants.

AMT: ammonium transporter, GS: glutamine synthetase, Gln: Glutamine, GOGAT: glutamate synthase, Glu: Glutamate, 2-OG: 2 oxoglutatate, TCA: Tricarboxylic acids, PGA: Phosphoglycerate, G3P: Glyceraldehyde 3-phosphate, Rubisco: Rubisco ribulose-1,5-bishosphate carboxylase.

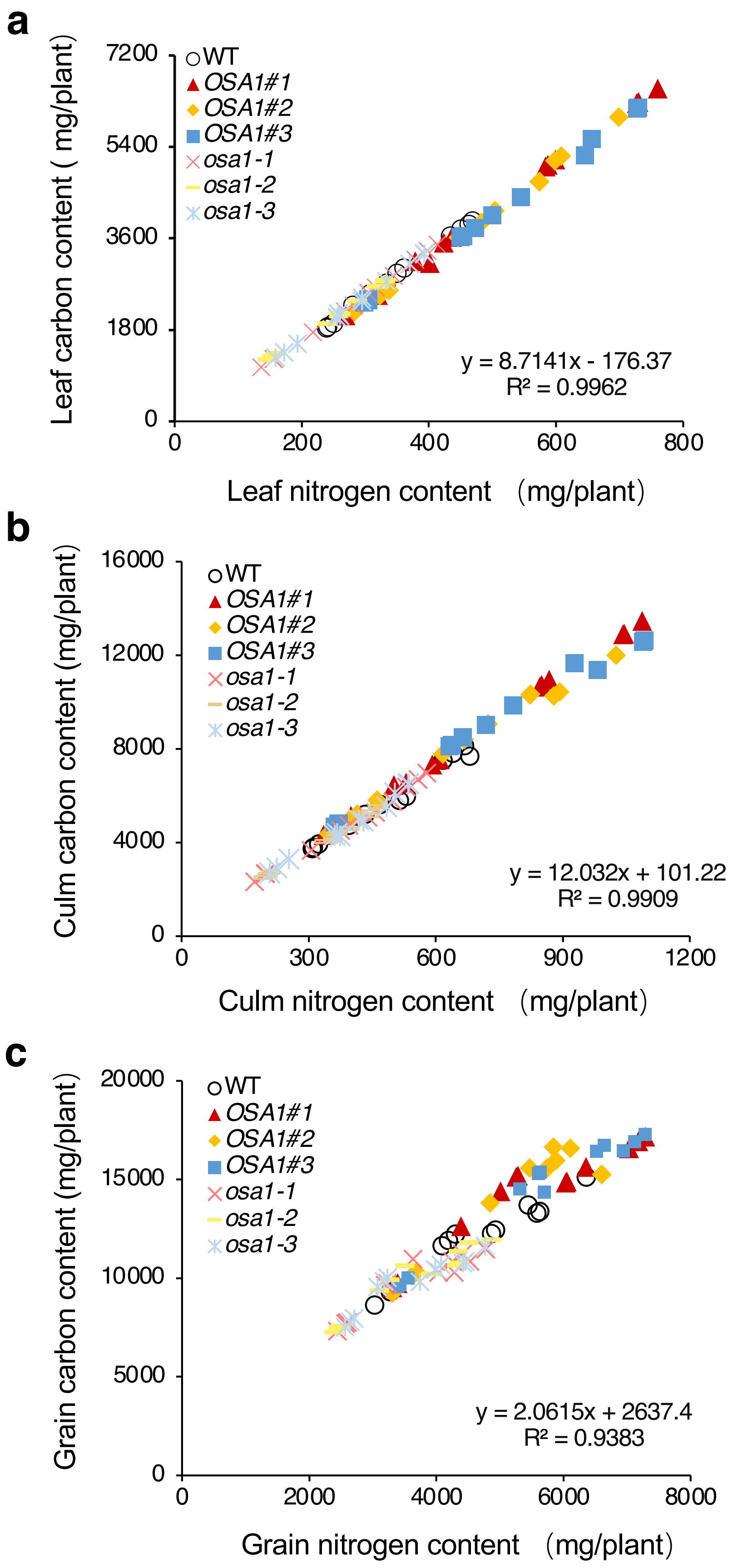

Supplementary Figure 13 Correlations of N and C content in WT, *OSA1*-oxs, and *osa1* mutant plants under various fertilisation levels in the field. Correlations between N content and C content in leaves (**a**), culms (**b**), and grain (**c**) of all rice genotypes harvested in the summer of 2017 at Nanjing-N.

Supplementary Table 1 Relative expression of *OSA* (*OSA2*, *OSA3*, *OSA4*, *OSA5*, *OSA6*, *OSA7*, *OSA8*, *OSA9*, *OSA10*) genes in rice leaves and roots of WT, *OSA1*-oxs, and *osa1* mutant lines. There were no significant difference among *OSA* isoforms. Differences were evaluated using the two-tailed Student's *t*-test. n.d. represents the gene expression that cannot be detected.

Root	*OSA2*	*OSA3*	*OSA4*	*OSA5*	*OSA6*	*OSA7*	*OSA8*	*OSA9*	*OSA10*
**WT**	1.00±0.08	1.00±0.26	n.d.	1.00±0.09	n.d.	1.00±0.18	1.06±0.08	1.27±0.16	1.10±0.19
OSA1#1	1.18±0.22	1.52±0.20	n.d.	0.93±0.11	n.d.	1.33±0.28	1.19±0.21	1.68±0.14	1.29±0.24
OSA1#2	1.23±0.25	1.31±0.18	n.d.	0.87±0.04	n.d.	1.22±0.11	1.16±0.10	1.71±0.36	1.23±0.13
OSA1#3	1.04±0.13	1.60±0.40	n.d.	1.18±0.20	n.d.	1.38±0.23	1.18±0.14	1.43±0.13	1.16±0.26
osa1-1	1.23±0.13	1.17±0.08	n.d.	0.98±0.16	n.d.	1.21±0.11	0.96±0.07	0.90±0.24	1.03±0.15
osa1-2	1.13±0.20	1.13±0.24	n.d.	0.92±0.23	n.d.	1.25±0.07	1.10±0.16	1.01±0.24	1.04±0.20
osa1-3	1.34±0.28	1.36±0.16	n.d.	1.05±0.25	n.d.	1.13±0.15	0.93±0.18	0.97±0.20	1.05±0.16

Leaf	*OSA2*	*OSA3*	*OSA4*	*OSA5*	*OSA6*	*OSA7*	*OSA8*	*OSA9*	*OSA10*
**WT**	1.00±0.06	1.10±0.17	n.d.	1.33±0.40	n.d.	1.14±0.10	1.16±0.07	n.d.	n.d.
OSA1#1	0.87±0.04	1.25±0.14	n.d.	1.64±0.44	n.d.	1.23±0.08	1.32±0.29	n.d.	n.d.
OSA1#2	1.09±0.05	1.44±0.21	n.d.	1.41±0.39	n.d.	1.33±0.16	1.15±0.16	n.d.	n.d.
OSA1#3	0.87±0.09	1.35±0.18	n.d.	1.32±0.39	n.d.	1.32±0.23	1.24±0.11	n.d.	n.d.
osa1-1	0.70±0.11	0.67±0.08	n.d.	1.64±0.34	n.d.	1.11±0.19	1.12±0.22	n.d.	n.d.
osa1-2	0.82±0.11	0.73±0.07	n.d.	1.18±0.47	n.d.	1.05±0.21	1.11±0.22	n.d.	n.d.
osa1-3	0.68±0.20	0.68±0.08	n.d.	1.55±0.73	n.d.	1.14±0.20	1.35±0.29	n.d.	n.d.

Supplementary Table 2 Gas-exchange properties of WT, *OSA1*-oxs, and mutant lines of hydroponically grown rice plants. Measurements were conducted at 400 µL L^{-1} CO_2. Light intensity was 1,000 µmol m^{-2} s^{-1}. Leaf temperature and relative humidity of leaf chamber were maintained at 24°C and 60–75% (Pa/Pa), respectively. Water use efficiency was calculated as the ratio between the CO_2 assimilation rate and transpiration rate. Differences were evaluated using the two-tailed Student's *t*-test (*$P < 0.05$; ** $P < 0.01$) ± SEs (n = 3).

	**WT**	***OSA1*#1**	***OSA1*#2**	***OSA1*#3**
**CO_2 assimilation rate (µmol m^{-2} s^{-1})**	11.61±0.53	14.62±0.87* (*P*=0.023)	14.88±1.39 (*P*=0.055)	14.59±0.78* (*P*=0.018)
**Stomatal conductance (mol m^{-2} s^{-1})**	0.41±0.05	0.73±0.02** (*P*=0.002)	0.88±0.08** (*P*=0.003)	0.83±0.07** (*P*=0.003)
**Ci (µL L^{-1})**	336.67±8.46	349.05±3.62 (*P*=0.175)	354.34±4.80 (*P*=0.090)	353.53±1.16 (*P*=0.073)
**Transpiration rate (mmol m^{-2} s^{-1})**	3.48±0.37	4.96±0.18* (*P*=0.012)	5.61±0.12** (*P*=0.003)	5.06±0.42* (*P*=0.027)
**Water use efficiency**	3.38±0.23	2.94±0.20 (*P*=0.150)	2.66±0.29 (*P*=0.075)	2.90±0.14 (*P*=0.089)

Supplementary Table 3 Agronomic traits and grain yields of WT, *OSA1*-oxs, and *osa1* mutant lines in the field (2016 Nanjing-S). Plant height, tiller number, panicle numbers, panicle length, spikelets, 1,000 grain weight, filled grains, and grain yield for plants grown in 2016 at Nanjing-S under 0 kg N/ha (N-N), 100 kg N/ha (L-N), 200 kg N/ha (M-N) and 300 kg N/ha (H-N) fertilisation. Differences were evaluated using one-way ANOVA. Values are the mean ± SEs (n ≥ 3). Letters indicates significant differences at $P < 0.05$ and the order starts with *OSA1*-oxs.

2016 Nanjing-S	Material	Plant height (cm)	Tiller number per hill	Panicles number per hill	Panicles length (cm)	spikelet number per panicle	1000 grain weight (g)	Filled grains rate (%)	Yield (kg/ha)
N-N (0 kg N/ha)	WT	81.3±1.1a	26.6±1.1b	24.9±0.8ab	17.2±0.5a	63.8±1.3b	26.1±0.5a	84.0±0.6a	6931±214b
	OSA1#1	83.0±1.4a	30.3±1.7a	27.4±1.4a	16.5±0.6a	69.1±1.4a	25.1±0.7a	86.7±1.3a	8191±423a
	OSA1#2	83.3±1.1a	33.8±1.3a	26.4±1.0a	16.8±0.2a	68.9±3.2a	25.4±0.6a	88.2±1.4a	8091±297a
	OSA1#3	81.7±0.8a	32.8±2.3a	26.3±2.3a	16.8±0.2a	69.5±2.1a	26.1±0.3a	86.1±2.4a	8157±721a
	osa1-1	77.0±3.1ab	25.1±0.6ab	22.5±0.7b	16.8±0.7a	58.4±1.5c	25.5±0.6a	84.6±0.8a	5644±176c
	osa1-2	78.0±3.1ab	25.3±0.7b	22.5±0.8b	16.5±0.6a	58.0±1.5c	25.0±0.5a	85.4±1.2a	5554±187c
	osa1-3	74.3±4.1b	24.6±0.7b	22.1±0.6b	16.3±0.5a	58.1±1.6c	25.6±1.0a	83.0±4.1a	5448±144c
L-N (100 kg N/ha)	WT	87.0±2.1a	29.8±1.2b	25.9±0.8b	19.5±0.9a	79.1±0.8b	25.8±0.4a	84.0±2.9a	8836±262b
	OSA1#1	91.7±5.7a	37.3±1.4a	30.8±1.1a	21.0±1.5a	83.5±2.0a	26.3±0.9a	87.4±0.4a	11757±414a
	OSA1#2	86.7±0.8a	38.0±0.9a	31.1±1.9a	18.9±0.6a	84.3±1.8a	26.1±0.4a	88.1±0.8a	12018±729a
	OSA1#3	85.3±0.8a	39.1±1.6a	32.3±1.7a	19.5±0.4a	85.0±1.9a	25.3±0.3a	87.4±2.2a	12088±632a
	osa1-1	84.7±3.6a	26.5±0.6c	22.9±0.7b	18.4±1.0a	74.1±0.9c	25.4±0.8a	84.7±1.2a	7268±217c
	osa1-2	84.0±2.5a	26.3±0.4c	22.8±0.5b	18.5±0.9a	74.8±0.7c	24.6±1.3a	83.7±1.8a	6987±149c
	osa1-3	83.3±2.5a	26.3±0.7c	22.9±0.4b	18.4±1.2a	75.5±1.0bc	25.6±0.6a	84.8±1.0a	7463±122c
M-N (200 kg N/ha)	WT	95.7±3.3a	33.5±2.0b	27.8±1.7b	20.0±1.3a	82.3±1.5b	25.1±1.3a	84.6±2.5a	9672±588b
	OSA1#1	96.7±1.5a	40.6±2.2a	33.3±1.2a	20.0±0.7a	88.6±1.0a	25.6±0.5a	86.9±3.4a	13060±454a
	OSA1#2	95.0±4.9a	41.6±2.0a	32.8±1.0a	19.7±1.5a	89.8±1.0a	25.9±0.3a	87.0±1.9a	13211±414a
	OSA1#3	96.0±2.4a	41.5±1.4a	32.6±0.9a	19.3±0.4a	90.4±2.2a	26.6±0.3a	85.8±2.6a	13393±370a
	osa1-1	92.3±4.3a	29.5±0.9c	25.1±0.9b	18.1±1.2a	76.5±1.5c	25.2±0.8a	83.9±2.3a	8097±294c
	osa1-2	91.7±3.3a	30.1±0.8c	25.8±0.9b	18.5±0.7a	76.0±1.5c	25.2±0.9a	83.8±2.2a	8243±272c
	osa1-3	92.3±2.2a	30.5±0.7c	25.9±0.6b	18.7±1.1a	75.5±1.6c	25.8±0.3a	83.4±3.1a	8370±190c
H-N (300 kg N/ha)	WT	95.3±2.0b	35.5±1.4b	30.0±0.8b	20.0±1.0a	84.5±1.9b	26.1±0.3a	83.7±1.0ab	11044±298b
	OSA1#1	104.0±3.2a	42.1±1.3a	34.9±2.2a	21.4±0.6a	92.5±1.7a	25.7±0.9a	85.6±1.8a	14120±883a
	OSA1#2	104.7±0.8a	43.8±2.2a	35.9±1.2a	21.5±1.1a	93.4±1.9a	25.7±0.6a	85.7±0.5a	14684±478a
	OSA1#3	102.7±0.4a	41.5±1.4a	36.0±1.6a	21.2±0.5a	93.1±1.3a	26.4±0.1a	86.4±0.7a	15205±677a
	osa1-1	95.7±1.6b	30.9±0.8c	26.8±0.6c	20.5±0.9a	78.6±1.8c	26.1±0.3a	83.2±1.0ab	9091±214c
	osa1-2	94.3±2.3b	30.6±1.0c	26.5±0.7c	20.1±0.2a	78.9±1.6c	26.1±0.1a	83.0±0.5ab	9008±228c
	osa1-3	96.0±1.2b	29.4±0.7c	25.5±0.5c	19.8±0.2a	78.5±1.3c	25.3±0.7a	81.6±2.7b	8232±146c

Supplementary Table 4 Agronomic traits and grain yields of WT, *OSA1*-oxs, and *osa1* mutant lines in the field (2017 Nanjing-N). Plant height, tiller number, panicle numbers, panicle length, spikelets, 1,000 grain weight, filled grains, and grain yield for plants grown in 2017 at Nanjing-N under 0 kg N/ha (N-N), 100 kg N/ha (L-N), 200 kg N/ha (M-N) and 300 kg N/ha (H-N) fertilisation. Differences were evaluated using one-way ANOVA. Values are the mean ± SEs (n ≥ 3). Letters indicates significant differences at $P < 0.05$ and the order starts with *OSA1*-oxs.

2017 Nanjing-N	Material	Plant height (cm)	Tiller number per hill	Panicles number per hill	Panicles length (cm)	spikelet number per panicle	1000 grain weight (g)	Filled grains rate (%)	Yield (kg/ha)
N-N (0 kgN/ha)	WT	81.7±1.5ab	25.0±1.2b	22.8±1.0bc	18.0±0.6a	64.7±2.9b	26.5±0.8a	81.3±1.5a	6340±265b
	OSA1#1	83.3±1.1a	30.4±1.9a	25.8±0.3a	17.3±1.2a	68.7±0.5ab	26.3±0.4a	80.8±5.0a	7517±98a
	OSA1#2	83.7±1.1a	34.4±2.1a	23.8±0.8ab	17.6±0.7a	71.7±1.8a	26.4±0.6a	82.6±0.9a	7422±240a
	OSA1#3	82.0±0.7ab	32.1±2.1a	24.5±1.6ab	17.6±0.4a	72.5±3.0a	26.6±0.7a	78.0±4.7a	7347±493a
	osa1-1	79.0±2.1ab	23.0±0.9b	20.2±1.1cd	17.5±0.6a	64.0±0.8b	26.5±0.8a	81.3±1.3a	5539±315b
	osa1-2	78.7±3.3ab	23.1±0.8b	20.3±0.9cd	17.1±0.7a	63.7±0.6b	26.4±1.0a	84.0±0.8a	5717±247b
	osa1-3	76.7±3.6b	22.8±0.6b	19.8±0.7d	17.3±0.2a	64.7±0.6b	26.4±0.4a	83.5±3.2a	5633±187b
L-N (100 kgN/ha)	WT	90.7±1.1a	29.5±1.6b	25.2±1.0b	19.2±2.1a	74.5±0.8b	25.9±0.4a	83.1±3.1a	8034±305b
	OSA1#1	95.7±3.2a	35.0±2.7a	27.8±0.9ab	19.4±1.9a	82.7±2.0a	26.6±0.7a	82.5±4.7a	10067±314a
	OSA1#2	90.7±3.6a	38.5±2.8a	28.5±1.3a	18.5±1.7a	84.0±2.2a	25.9±0.8a	85.4±3.7a	10533±499a
	OSA1#3	89.3±4.3a	36.8±2.2a	29.0±2.1a	19.2±1.8a	85.0±3.6a	25.6±0.7a	81.0±6.0a	10176±743a
	osa1-1	88.3±2.9a	24.9±0.6b	22.0±1.0c	19.2±0.9a	68.8±1.1c	26.1±0.8a	85.7±1.3a	6747±300c
	osa1-2	88.0±2.5a	24.8±0.8b	21.8±0.4c	18.5±1.1a	69.0±1.0c	26.6±0.4a	84.8±0.4a	6762±136c
	osa1-3	88.3±2.5a	24.9±0.7b	21.7±0.6c	18.9±0.8a	69.2±0.9c	26.4±1.3a	84.9±2.4a	6687±189c
M-N (200 kgN/ha)	WT	92.7±5.1a	30.3±1.2c	26.2±1.6b	19.9±2.7a	79.2±2.1b	25.6±0.7a	86.7±0.8a	9177±556b
	OSA1#1	93.7±1.8a	35.9±1.4b	31.3±1.8a	20.0±1.9a	87.5±1.6a	26.1±0.7a	81.4±5.5a	11615±660a
	OSA1#2	92.0±4.4a	39.1±1.8a	31.5±2.1a	19.5±2.7a	90.2±1.9a	26.0±0.7a	80.2±4.8a	11785±797a
	OSA1#3	93.0±3.5a	38.5±1.1ab	30.8±1.1a	19.7±1.5a	90.0±1.4a	25.8±1.0a	82.8±5.3a	11834±426a
	osa1-1	91.7±2.7a	26.9±0.7d	23.2±1.3b	19.6±0.5a	73.3±2.0c	24.9±0.7a	84.6±1.0a	7139±413c
	osa1-2	89.0±2.8a	26.3±0.6d	22.7±0.7b	19.1±1.3a	73.5±2.0c	26.1±1.1a	84.1±2.7a	7298±235c
	osa1-3	90.7±3.3a	26.9±0.7d	23.7±0.6b	19.6±1.0a	74.2±1.8c	26.9±.0.8a	81.2±8.5a	7648±197c
H-N (300 kgN/ha)	WT	97.3±6.7a	31.6±0.8b	30.0±0.9b	20.2±1.5a	82.2±2.8b	25.4±0.7b	82.0±1.4ab	10230±305b
	OSA1#1	106.0±5.8a	38.1±2.7a	32.5±1.8ab	21.6±2.0a	92.7±2.3a	25.4±0.7b	86.2±1.7ab	13119±729a
	OSA1#2	106.7±4.6a	40.0±1.2a	34.7±1.0a	21.7±1.5a	95.3±2.8a	25.8±0.4ab	78.1±6.8b	13273±370a
	OSA1#3	104.7±5.2a	40.1±0.8a	34.2±0.8a	21.4±1.7a	96.8±1.5a	25.9±0.7ab	82.9±0.6ab	14162±319a
	osa1-1	97.7±6.4a	29.5±0.6b	26.5±0.8c	20.6±1.2a	78.2±1.3b	26.9±0.6ab	83.9±0.9ab	9315±277b
	osa1-2	96.0±4.4a	29.4±0.4b	26.5±0.4c	20.4±1.8a	78.0±1.6b	26.4±0.6ab	87.0±3.7a	9450±133b
	osa1-3	98.0±6.3a	28.9±0.6b	27.0±0.7c	20.0±1.6a	77.8±1.3b	27.7±1.2a	82.3±0.4ab	9558±245b

Supplementary Table 5 Agronomic traits and grain yields of WT , *OSA1*-oxs, and *osa1* mutant lines in the field (2017 Fengyang). Plant height, tiller number, panicle numbers, panicle length, spikelets, 1,000 grain weight, filled grains, and grain yield for plants grown in 2017 at Fengyang under 0 kg N/ha (N-N), 100 kg N/ha (L-N), 200 kg N/ha (M-N) and 300 kg N/ha (H-N) fertilisation. Differences were evaluated using one-way ANOVA. Values are the mean ± SEs (n ≥ 3). Letters indicates significant differences at $P < 0.05$ and the order starts with *OSA1*-oxs.

2017 Fengyang	Material	Plant height (cm)	Tiller number per hill	Panicles number per hill	Panicles length (cm)	spikelet number per panicle	1000 grain weight (g)	Filled grains rate (%)	Yield (kg/ha)
N-N (0 kgN/ha)	WT	72.0±1.4a	24.5±1.5b	23.5±0.9a	16.8±0.9a	63.0±1.3b	25.8±0.4a	86.5±2.1a	6588±247b
	OSA1#1	73.7±1.1a	28.7±1.4a	25.5±1.2a	16.2±1.7a	69.0±1.6a	25.8±0.8a	86.1±5.9a	7798±381a
	OSA1#2	73.7±1.8a	29.8±1.6a	24.5±0.5a	16.5±1.1a	69.4±1.0a	26.3±0.2a	83.3±1.5a	7404±137a
	OSA1#3	72.3±0.4a	30.0±1.7a	24.9±0.8a	16.5±1.3a	71.5±1.2a	25.7±1.3a	87.1±6.3a	7935±269a
	osa1-1	69.7±1.8a	22.8±1.1b	20.5±0.9b	16.4±0.8a	58.5±1.3c	25.9±0.5a	84.3±2.9a	5212±235c
	osa1-2	70.0±1.4a	22.3±0.6b	20.5±0.7b	15.9±0.5a	57.5±1.5c	26.1±0.6a	84.3±0.2a	5162±183c
	osa1-3	69.7±2.2a	22.7±1.0b	20.3±0.6b	16.0±1.4a	57.5±1.7c	26.8±0.7a	85.0±1.9a	5292±165c
L-N (100 kgN/ha)	WT	87.0±1.9a	28.2±0.9b	23.8±1.2b	18.7±1.2a	72.1±1.3c	25.7±0.3a	86.0±3.0ab	7545±378b
	OSA1#1	91.7±3.5a	39.2±2.3a	27.9±1.8a	19.7±0.8a	80.4±1.5ab	26.2±0.7a	87.1±4.2a	10177±645a
	OSA1#2	87.0±4.2a	39.0±4.2a	28.8±0.9a	18.6±1.2a	79.9±1.3b	27.2±1.3a	79.4±1.7b	9884±301a
	OSA1#3	85.7±5.0a	39.5±2.7a	28.1±1.1a	18.7±1.5a	84.0±1.4a	26.4±0.9a	81.8±1.9ab	10144±382a
	osa1-1	84.7±2.7a	25.0±1.3b	21.1±0.7b	17.3±1.5a	67.0±1.5d	25.4±0.9a	83.2±0.3ab	5955±193c
	osa1-2	84.3±3.3a	25.3±0.5b	21.3±0.6b	17.4±1.3a	66.0±1.3d	26.2±0.9a	79.5±2.2b	5809±163c
	osa1-3	84.7±3.6a	25.3±0.8b	21.4±0.5b	17.6±1.4a	68.0±1.6d	26.1±0.7a	84.5±2.2ab	6386±147c
M-N (200 kgN/ha)	WT	87.7±4.5a	30.3±1.8b	25.4±0.8b	19.0±1.4ab	76.1±1.1c	26.4±0.9ab	82.6±2.2a	8400±250b
	OSA1#1	88.7±1.6a	40.3±1.7a	29.8±1.3a	19.7±0.6ab	84.6±1.2b	24.8±0.4b	88.9±2.9a	11044±490a
	OSA1#2	87.0±3.7a	42.0±1.7a	30.4±0.7a	20.1±0.7a	84.1±0.9b	25.3±1.0ab	85.3±3.9a	10995±242a
	OSA1#3	88.0±3.2a	40.2±2.3a	29.3±0.7a	19.3±0.5ab	88.6±1.8a	25.8±0.8ab	86.3±6.2a	11494±272a
	osa1-1	83.0±4.6a	27.3±1.5b	22.3±0.5c	17.7±1.0b	71.0±1.0d	26.9±0.6a	80.7±1.5a	6822±161c
	osa1-2	85.3±2.9a	27.0±0.8b	22.0±0.5c	17.8±0.2ab	71.4±0.8d	25.1±0.4ab	84.4±0.7a	6615±149c
	osa1-3	83.3±3.9a	28.0±0.7b	22.8±0.4c	18.5±0.9ab	71.8±0.7d	25.6±0.8ab	83.0±2.4a	6909±119c
H-N (300 kgN/ha)	WT	93.0±3.9ab	32.0±1.1b	28.0±0.5b	19.4±1.2a	79.0±1.8b	25.1±0.6b	83.9±2.3a	9267±150b
	OSA1#1	101.3±4.5a	42.2±1.4a	31.3±1.5a	21.4±2.6a	88.9±2.0a	26.0±1.4ab	84.3±2.5a	12117±569a
	OSA1#2	101.7±2.5a	42.0±1.9a	31.6±0.9a	20.9±1.3a	89.6±1.2a	24.9±0.5b	86.9±9.0a	12233±349a
	OSA1#3	99.7±2.9ab	42.3±1.9a	31.6±1.3a	20.9±1.7a	93.3±2.5a	27.1±0.4a	79.9±4.6a	12742±533a
	osa1-1	93.3±3.6ab	30.8±0.8b	24.9±0.4c	19.7±1.3a	73.4±1.8c	25.8±0.4ab	88.1±0.5a	8263±141c
	osa1-2	91.7±1.6b	30.8±0.7b	24.8±0.6c	20.1±1.9a	73.5±1.9c	25.1±0.1b	82.2±0.9a	7477±170c
	osa1-3	93.7±3.9ab	31.3±0.8b	24.5±0.5c	19.6±1.9a	73.8±1.4c	25.1±0.8b	84.8±2.3a	7659±141c

Supplementary Table 6 Agronomic traits and grain yields of WT , *OSA1*-oxs, and *osa1* mutant lines in the field (2016 Hainan). Plant height, tiller number, panicle numbers, panicle length, spikelets, 1,000 grain weight, filled grains, and grain yield for plants grown in 2016 at Hainan under 0 kg N/ha (N-N), 100 kg N/ha (L-N), 200 kg N/ha (M-N) and 300 kg N/ha (H-N) fertilisation. Differences were evaluated using one-way ANOVA. Values are the mean ± SEs (n ≥ 3). Letters indicates significant differences at $P < 0.05$ and the order starts with *OSA1*-oxs.

2016 Hainan	Material	Plant Height (cm)	Tiller number per hill	Panicles number per hill	Panicles length (cm)	spikelet number per panicle	1000 grain weight (g)	Filled grains rate (%)	Yield (kg/ha)
N-N (0 kgN/ha)	WT	67.2±2.1ab	16.8±0.7b	14.5±0.5c	16.4±0.4a	60.5±2.6bc	25.1±0.2a	78.6±2.5a	3446±127c
	OSA1#1	70.8±1.6a	18.6±0.8ab	16.0±0.6abc	15.9±0.7a	65.5±2.2ab	24.5±1.3a	80.6±2.4a	4112±164b
	OSA1#2	70.2±1.1ab	20.2±1.0ab	17.3±0.4a	16.8±0.8a	67.7±2.4a	24.3±0.9a	81.7±1.0a	4607±104a
	OSA1#3	71.2±2.4a	20.0±2.1ab	16.8±0.9ab	17.1±0.8a	67.5±1.6a	25.5±0.7a	81.4±1.8a	4663±237a
	osa1-1	65.6±2.9ab	17.8±1.2ab	14.8±0.8c	16.8±1.4a	51.0±1.4d	24.7±0.7a	79.6±2.4a	2948±155d
	osa1-2	65.4±2.3ab	20.0±0.8ab	15.1±0.6bc	16.7±0.8a	57.5±3.1cd	24.4±0.9a	78.4±3.1a	3313±121cd
	osa1-3	64.0±3.0b	20.6±1.0a	15.0±0.6bc	16.4±1.0a	56.5±2.9cd	24.4±0.8a	79.0±3.7a	3248±138cd
L-N (100 kgN/ha)	WT	77.2±3.0a	31.8±1.7b	21.9±0.9b	19.5±1.6a	69.0±4.0bc	25.5±0.3a	80.3±2.4a	6160±251b
	OSA1#1	80.4±1.4a	39.0±0.6a	25.0±0.4a	21.1±0.8a	77.2±3.7ab	25.7±0.5a	81.4±3.0a	8032±130a
	OSA1#2	82.2±2.0a	38.0±2.3a	25.4±0.6a	19.2±0.7a	76.5±2.9ab	26.2±1.0a	82.1±1.8a	8309±209a
	OSA1#3	82.0±2.7a	40.8±0.9a	25.0±0.3a	19.9±1.1a	79.2±3.1a	25.5±0.7a	82.5±0.8a	8298±95a
	osa1-1	74.2±4.6a	30.6±2.1b	20.1±0.9bc	16.3±0.3b	64.0±2.5c	24.4±0.8a	80.7±5.0a	5058±224c
	osa1-2	75.8±3.9a	29.4±1.8b	19.3±0.7c	16.3±1.5b	61.3±3.3c	24.4±1.2a	79.3±1.5a	4547±164c
	osa1-3	75.4±3.7a	29.8±1.0b	17.4±0.6d	16.3±0.4b	65.8±2.0c	25.1±0.5a	82.8±1.6a	4736±165c
M-N (200 kgN/ha)	WT	83.8±2.3a	33.8±1.4b	24.3±0.8b	19.1±1.2a	73.8±3.1bc	24.7±0.5a	81.0±2.6a	7119±228c
	OSA1#1	86.2±2.4a	42.6±1.9a	27.6±0.7a	20.1±1.3a	80.3±2.7ab	25.1±0.5a	83.5±2.4a	9286±235ab
	OSA1#2	85.6±1.9a	42.4±2.0a	28.3±0.6a	19.4±1.2a	80.5±2.8ab	24.2±0.5a	82.9±2.1a	9087±203b
	OSA1#3	85.0±2.1a	43.4±1.2a	27.4±0.5a	19.1±0.9a	85.5±3.6a	24.5±0.6a	84.4±2.5a	9662±174a
	osa1-1	83.4±3.0a	34.8±0.8b	20.6±0.6c	18.7±0.7a	65.7±2.9d	24.1±1.8a	83.5±1.0a	5431±168de
	osa1-2	80.0±4.9a	31.6±1.0bc	21.1±0.9c	18.3±0.6a	67.5±2.8cd	25.0±0.2a	81.8±1.3a	5814±245d
	osa1-3	81.6±3.4a	29.2±2.2c	20.0±0.6c	18.6±1.8a	64.5±2.2d	24.6±0.7a	80.2±1.7a	5074±162e
H-N (300 kgN/ha)	WT	89.0±2.4ab	33.8±3.0b	26.8±0.5b	20.1±1.2a	77.3±2.6bc	24.4±.0.8a	84.1±2.1a	8469±153c
	OSA1#1	91.0±2.4a	40.0±1.5a	29.1±0.6a	21.1±0.4a	83.3±2.2ab	25.2±0.3a	81.6±5.4a	9933±200b
	OSA1#2	92.2±2.1a	40.8±1.0a	29.9±0.5a	21.5±0.8a	85.2±2.0a	25.5±0.7a	83.1±1.5a	10723±184a
	OSA1#3	91.2±1.6a	44.0±1.7a	29.3±1.0a	21.1±1.4a	85.7±1.9a	24.7±0.6a	84.0±0.9a	10361±341ab
	osa1-1	82.0±1.4b	34.0±1.3b	23.5±0.7c	20.4±1.3a	71.5±1.6c	25.4±1.4a	82.5±0.9a	7017±200de
	osa1-2	85.2±3.5ab	34.6±1.6b	23.9±0.7c	20.2±0.6a	72.7±2.5c	26.0±0.3a	82.1±0.9a	7368±229d
	osa1-3	85.6±4.5ab	33.8±0.8b	23.4±0.7c	20.4±0.6a	73.2±3.8c	24.3±0.6a	80.8±2.1a	6684±200e

Supplementary Table 7 List of primers used in this study.

Gene name	Forward (F)/ Reverse (R)	Sequences (5'–3')	Application
	F	AGGTCGACTCTAGAGGATCCTAGGGTCAGCATAGCAGT	
OSA1	R	CTCAGATCTACCATGGTACCGGAGGCGACCTCCCACACCT	Transformation
	F	GGAACTGGTATGGTCAAGGC	
OsActin	R	AGTCTCATGGATAACCGCAG	RT-PCR
	F	TGGCTGGCATGGATGTTCTT	
OSA1	R	TTCCTAGACGACGCCCTGTT	RT-PCR
	F	TTATGGTTGGGATGGGACA	
OsActin	R	AGCACGGCTTGAATAGCG	quantitative RT-PCR
	F	TCAAATGCTAGCTGCACCAC	
OsGAPDH	R	GCAGTGATGGCATGAACAGT	quantitative RT-PCR
	F	GTGTTTGGGTTTATGCTGCT	
OSA1	R	GTATCCACCCAGCACAACTC	quantitative RT-PCR
	F	ACTGAGCCAGGCCTTAGTGT	
OSA2	R	TCATTGAGGATGGCAATGAT	quantitative RT-PCR
	F	GAGGAGAGGGAGCTCAAGTG	
OSA3	R	CACAACCGATTCTACATGCC	quantitative RT-PCR
	F	TCAGCATCGTCACCTTCTTC	
OSA4	R	CTCTTCCCGTAGTCCAGCTC	quantitative RT-PCR
	F	TCTGGCTCTACAGCATCGTC	
OSA5	R	TCCCGTAATCCTTCTTGCTC	quantitative RT-PCR
	F	TCAGCGTGGTGACCTACTTC	
OSA6	R	CCCGTAGTCGTTCTTGTTCA	quantitative RT-PCR
	F	GGGCTGGGCTGGCGTTATCT	
OSA7	R	TTGAAGAGCGTGTTGGAGGC	quantitative RT-PCR
	F	TCAACCAAATGGCTGAAGAG	
OSA8	R	CCACAGATTCCACCTTTCCT	quantitative RT-PCR
	F	GTCCTTCCTCGAGAGACCTG	
OSA9	R	GCGTAGAACACCAGGCTGTA	quantitative RT-PCR
	F	GCGGATGAAGAACTACACCA	
OSA10	R	CTTGGAGATGGTCATGATGG	quantitative RT-PCR
	F	TGTTTCTCCTCATCCCTGC	
OsGS1.2	R	TCACAGTCCTCGCTTTGC	quantitative RT-PCR
	F	GGAGAGGTCATGCCTGGTCAGT	
OsGS2	R	ACTACACCAGCCTGCTCCGTTA	quantitative RT-PCR
	F	GTGCAGCCTGTTGCAGCATAAA	
OsNADH-GOGAT1	R	CGGCATTTCACCATGCAAATC	quantitative RT-PCR
	F	CCTGTCGAAGGATGATGAAGGTGAAACC	
OsNADH-GOGAT2	R	TGCATGGCCCTACTATCTTCGCATCA	quantitative RT-PCR
	F	GGTTTCTCTCCCTCTCCGAT	
OsAMT1.1	R	CCACCTTCACACCACACATT	quantitative RT-PCR
	F	GAAAGCCTGTGGAAACGCA	
OsGRF4	R	CAACGCCGAGCCAAATGAG	quantitative RT-PCR
