# Supplementary material for: Plasma membrane H+-ATPase overexpression increases rice yield via simultaneous enhancement of nutrient uptake and photosynthesis
Source: Nat Commun. 2021 Feb 2;12:735. doi: 10.1038/s41467-021-20964-4 (PMC7854686; doi:10.1038/s41467-021-20964-4)
Supplement: Supplementary file 3 — Descriptions of Additional Supplementary Files [file 41467_2021_20964_MOESM3_ESM.pdf]

Descriptions of Additional Supplementary Files

Supplementary Data 1

Description: Significantly enriched GO terms in the DEGs up-regulated by the overexpression of OSA1 and the DEGs down-regulated by the knockout mutation of osa1.Differences were evaluated using the ultra-geometric test(FDR< 0.05).

Supplementary Data 2

Description: Significantly enriched GO terms in the DEGs up-regulated in both leaves and roots of OSA1-ox, and down-regulated in both leaves and roots of osa1. Differences were evaluated using the ultra-geometric test (FDR< 0.05).

Supplementary Data 3

Description: Significantly enriched GO terms in the DEGs down-regulated by the overexpression of OSA1 and also up-regulated by the knockout mutation of osa1 in leaves and roots of rice. Differences were evaluated using the ultra-geometric test(FDR< 0.05).
