# Supplementary material for: Plasma membrane H+-ATPase overexpression increases rice yield via simultaneous enhancement of nutrient uptake and photosynthesis
Source: Nat Commun. 2021 Feb 2;12:735. doi: 10.1038/s41467-021-20964-4 (PMC7854686; doi:10.1038/s41467-021-20964-4)
Supplement: Supplementary file 7 — Reporting Summary [file 41467_2021_20964_MOESM7_ESM.pdf]

nature research

Corresponding author(s): Toshinori Kinoshita

Last updated by author(s): Dec 23, 2020

Reporting Summary

Nature Research wishes to improve the reproducibility of the work that we publish. This form provides structure for consistency and transparency in reporting. For further information on Nature Research policies, see our Editorial Policies and the Editorial Policy Checklist.

Statistics

For all statistical analyses, confirm that the following items are present in the figure legend, table legend, main text, or Methods section.

n/a	Confirmed	
☐	☒	The exact sample size (*n*) for each experimental group/condition, given as a discrete number and unit of measurement
☐	☒	A statement on whether measurements were taken from distinct samples or whether the same sample was measured repeatedly
☐	☒	The statistical test(s) used AND whether they are one- or two-sided<br>*Only common tests should be described solely by name; describe more complex techniques in the Methods section.*
☐	☒	A description of all covariates tested
☒	☐	A description of any assumptions or corrections, such as tests of normality and adjustment for multiple comparisons
☐	☒	A full description of the statistical parameters including central tendency (e.g. means) or other basic estimates (e.g. regression coefficient) AND variation (e.g. standard deviation) or associated estimates of uncertainty (e.g. confidence intervals)
☐	☒	For null hypothesis testing, the test statistic (e.g. *F*, *t*, *r*) with confidence intervals, effect sizes, degrees of freedom and *P* value noted<br>*Give P values as exact values whenever suitable.*
☒	☐	For Bayesian analysis, information on the choice of priors and Markov chain Monte Carlo settings
☒	☐	For hierarchical and complex designs, identification of the appropriate level for tests and full reporting of outcomes
☒	☐	Estimates of effect sizes (e.g. Cohen's *d*, Pearson's *r*), indicating how they were calculated

Our web collection on statistics for biologists contains articles on many of the points above.

Software and code

Policy information about availability of computer code

Data collection	Microsoft Excel 2006. Sequencing reads were undertaken with the NextSeq 500 system (Illumina) sequencer.
Data analysis	Sigma Plot v12.5, SPSS v18.0, R language 3.6.2, Microsoft Excel 2006, EdgeR software package, Degust Ver. 3.1.0 , Plant Transcriptional Regulatory Map ,GO category ,Next Seq 500 pipeline and Bowtie statistical softwares were used for statistical analysis.

For manuscripts utilizing custom algorithms or software that are central to the research but not yet described in published literature, software must be made available to editors and reviewers. We strongly encourage code deposition in a community repository (e.g. GitHub). See the Nature Research guidelines for submitting code & software for further information.

Data

Policy information about availability of data

All manuscripts must include a data availability statement. This statement should provide the following information, where applicable:

- Accession codes, unique identifiers, or web links for publicly available datasets
- A list of figures that have associated raw data
- A description of any restrictions on data availability

The authors declare that the data supporting the findings of this study are available within the paper and the Supplementary Information.

Field-specific reporting

Life sciences study design

All studies must disclose on these points even when the disclosure is negative.

Sample size	Required experimental sample sizes were estimated based on our past experience performing similar experiments including field test.
Data exclusions	The results were excluded from the analysis if the positive control experiments were not successful.
Replication	All experiments were successfully repeated at least three times, and we have stated it in figure legends .
Randomization	Plants of equal initial sizes were randomly assigned to the treatment and control groups.
Blinding	Analysis were performed in a manner blinded to treatment assignment in all experiments.

Reporting for specific materials, systems and methods

We require information from authors about some types of materials, experimental systems and methods used in many studies. Here, indicate whether each material, system or method listed is relevant to your study. If you are not sure if a list item applies to your research, read the appropriate section before selecting a response.

Materials & experimental systems

n/a	Involved in the study	
☐	☒	Antibodies
☒	☐	Eukaryotic cell lines
☒	☐	Palaeontology and archaeology
☒	☐	Animals and other organisms
☒	☐	Human research participants
☒	☐	Clinical data
☒	☐	Dual use research of concern

Methods

n/a	Involved in the study	
☒	☐	ChIP-seq
☒	☐	Flow cytometry
☒	☐	MRI-based neuroimaging

Antibodies

Antibodies used	PM H+-ATPase was detected with anti-H+-ATPase antibody as described previously(Hayashi,et.al 2010 Plant Cell Physiol.). Actin as the internal control protein was detected with anti-actin antibodies (1:3,000 dilution, Sigma-Aldrich, Cat#057M4548).
Validation	Anti-H+-ATPase antibody is a polyclonal antibody from rabbit with high affinity to PM H+-ATPase catalytic domain. Anti-actin antibody is a monoclonal antibody from mouse with high affinity to plant actin as an internal control protein .Both antibodies can be applied in Western Blot for specifically detecting PM H+-ATPase and actin, respectively.
