# Supplementary material for: Plasma membrane H+-ATPase overexpression increases rice yield via simultaneous enhancement of nutrient uptake and photosynthesis
Source: Nat Commun. 2021 Feb 2;12:735. doi: 10.1038/s41467-021-20964-4 (PMC7854686; doi:10.1038/s41467-021-20964-4)
Supplement: Supplementary file 8 — Source Data [file 41467_2021_20964_MOESM8_ESM.zip › p-values.docx]

**1.**The exact *p* values in **FIG.2** are 0.0241 (WT vs. *OSA1#1*) in root, 0.0292 (WT vs. *OSA1#2*) in root, 0.0203 (WT vs. *OSA1#3*) in root, 0.0124 (WT vs. *OSA1#1*) in shoot, 0.0173 (WT vs. *OSA1#2*) in shoot, and 0.0092 (WT vs. *OSA1#3*) in shoot for **b**; 3.2×10^−6^ (WT vs. *OSA1#1*) in roots, 4.1×10^−6^ (WT vs. *OSA1#2*) in roots, 7.7×10^−4^ (WT vs. *OSA1#3*) in roots, 7.9×10^−4^ (WT vs. *OSA1#1*) in leaves, 0.0026 (WT vs. *OSA1#2*) in leaves, and 2.8×10^−6^ (WT vs. *OSA1#3*) in leaves for **c**; 0.0458 (WT vs. *OSA1#1*) in roots, 0.0025 (WT vs. *OSA1#2*) in roots, 0.0068 (WT vs. *OSA1#3*) in roots, 0.0021 (WT vs. *OSA1#1*) in leaves, 0.0464 (WT vs. *OSA1#2*) in leaves, and 0.0329 (WT vs. *OSA1#3*) in leaves for **d**; 0.0037 (WT vs. *OSA1#1*) in roots, 0.0023 (WT vs. *OSA1#2*) in roots, 0.0216 (WT vs. *OSA1#3*) in roots, 0.0022 (WT vs. *OSA1#1*) in leaves, 0.0050 (WT vs. *OSA1#2*) in leaves, and 0.0002 (WT vs. *OSA1#3*) in leaves for **e**; 3.7×10 ^−7^ (WT vs. *OSA1#1*) in 0.5 mM NH_4_^+^, 8.6×10 ^−7^ (WT vs. *OSA1#2*) in 0.5 mM NH_4_^+^, 7.9×10^−8^(WT vs. *OSA1#3*) in 0.5 mM NH_4_^+^, 5.4×10^−5^ (WT vs. *OSA1#1*) in 1 mM NH_4_^+^, 2.2×10 ^−5^ (WT vs. *OSA1#2*) in 1 mM NH_4_^+^, 1.3×10 ^−4^(WT vs. *OSA1#3*) in 1 mM NH_4_^+^, 4.2×10^−6^(WT vs. *OSA1#1*) in 2 mM NH_4_^+^, 8.3×10^−6^(WT vs. *OSA1#2*) in 2 mM NH_4_^+^, 2.4×10^−7^(WT vs. *OSA1#3*) in 2 mM NH_4_^+^, 9.2×10^−4^(WT vs. *OSA1#1*) in 4 mM NH_4_^+^, 0.0013 (WT vs. *OSA1#2*) in 4 mM NH_4_^+^, 1.0×10^−4^(WT vs. *OSA1#3*) in 4 mM NH_4_^+^, 1.3×10^−6^ (WT vs. *OSA1#1*) in 8 mM NH_4_^+^, 1.5×10^−7^ (WT vs. *OSA1#2*) in 8 mM NH_4_^+^, and 1.8×10^−7^(WT vs. *OSA1#3*) in 8 mM NH_4_^+^ for **f**; 0.0138 (WT vs. *OSA1#1*) in roots, 0.0076 (WT vs. *OSA1#2*) in roots, 0.0049 (WT vs. *OSA1#3*) in roots, 0.0208 (WT vs. *OSA1#1*) in leaves, 0.0242 (WT vs. *OSA1#2*) in leaves, and 0.0015 (WT vs. *OSA1#3*) in leaves for **g**; 0.0079 (WT vs. *OSA1#1*) in roots, 0.0143 (WT vs. *OSA1#2*) in roots, 0.0119 (WT vs. *OSA1#3*) in roots, 0.0061 (WT vs. *OSA1#1*) in leaves, 0.0098 (WT vs. *OSA1#2*) in leaves, and 0.0009 (WT vs. *OSA1#3*) in leaves for **h**.

**2.**The exact *p* values in **FIG.3** are 0.0038 (WT vs. *osa1-1*) in root, 0.0010 (WT vs. *osa1-2*) in root, 0.0009 (WT vs. *osa1-3*) in root, 0.0008 (WT vs. *osa1-1*) in shoot, 0.0003 (WT vs. *osa1-2*) in shoot, and 0.0008 (WT vs. *osa1-3*) in shoot for **b**; 2.8×10^−4^ (WT vs. *osa1-1*) in roots, 2.1×10^−4^ (WT vs. *osa1-2*) in roots, 2.3×10^−4^ (WT vs. *osa1-3*) in roots, 3.0×10^−7^ (WT vs. *osa1-1*) in leaves, 8.6×10^−7^ (WT vs. *osa1-2*) in leaves, and 6.0×10^−7^ (WT vs. *osa1-3*) in leaves for **c**; 0.0021 (WT vs. *osa1-1*) in roots, 0.0427 (WT vs. *osa1-2*) in roots, 9.2×10^−5^ (WT vs. *osa1-3*) in roots, 0.0087 (WT vs. *osa1-1*) in leaves, 0.0255 (WT vs. *osa1-2*) in leaves, and 0.0202 (WT vs. *osa1-3*) in leaves for **d**; 0.0401 (WT vs. *osa1-1*) in roots, 0.0096 (WT vs. *osa1-2*) in roots, 0.0146 (WT vs. *osa1-3*) in roots, 0.0239 (WT vs. *osa1-1*) in leaves, 0.0081 (WT vs. *osa1-2*) in leaves, and 0.0078 (WT vs. *osa1-3*) in leaves for **e**; 6.0×10 ^−8^ (WT vs. *osa1-1*) in 0.5 mM NH_4_^+^, 4.0×10 ^−7^ (WT vs. *osa1-2*) in 0.5 mM NH_4_^+^, 1.7×10^−8^ (WT vs. *osa1-3*) in 0.5 mM NH_4_^+^, 0.0029 (WT vs. *osa1-1*) in 1 mM NH_4_^+^, 5.4×10^−4^ (WT vs. *osa1-2*) in 1 mM NH_4_^+^, 0.0028 (WT vs. *osa1-3*) in 1 mM NH_4_^+^, 3.8×10^−6^ (WT vs. *osa1-1*) in 2 mM NH_4_^+^, 7.9×10^−6^ (WT vs. *osa1-2*) in 2 mM NH_4_^+^, 1.9×10^−5^ (WT vs. *osa1-3*) in 2 mM NH_4_^+^, 3.7×10^−4^ (WT vs. *osa1-1*) in 4 mM NH_4_^+^, 1.5×10^−4^ (WT vs. *osa1-2*) in 4 mM NH_4_^+^, 2.3×10^−5^ (WT vs. *osa1-3*) in 4 mM NH_4_^+^, 4.7×10^−4^ (WT vs. *osa1-1*) in 8 mM NH_4_^+^, 5.6×10^−4^ (WT vs. *osa1-2*) in 8 mM NH_4_^+^, and 6.2×10^−5^ (WT vs. *osa1-3*) in 8 mM NH_4_^+^ for **f**; 0.0012 (WT vs. *osa1-*) in roots, 4.1×10^−4^ (WT vs. *osa1-2*) in roots, 4.0×10^−4^ (WT vs. *osa1-3*) in roots, 3.0×10^−4^ (WT vs. *osa1-1*) in leaves, 1.3×10^−4^ (WT vs. *osa1-2*) in leaves, and 3.1×10^−4^ (WT vs. *osa1-3*) in leaves for **g**; 0.0025 (WT vs. *osa1-1*) in roots, 7.5×10^−4^ (WT vs. *osa1-2*) in roots, 8.1×10^−4^ (WT vs. *osa1-3*) in roots, 6.3×10^−4^ (WT vs. *osa1-1*) in leaves, 3.2×10^−4^ (WT vs. *osa1-2*) in leaves, and 5.8×10^−4^ (WT vs. *osa1-3*) in leaves for **h**.

**3.**The exact *p* values in **FIG.4** are 0.7202 (WT vs. *OSA1#1*) in DK, 0.8948 (WT vs. *OSA1#2*) in DK, 0.9860 (WT vs. *OSA1#3*) in DK, 0.0084 (WT vs. *OSA1#1*) in RL+BL, 0.0121 (WT vs. *OSA1#2*) in RL+BL, 0.0029 (WT vs. *OSA1#3*) in RL+BL, 0.0539 (WT vs. *OSA1#1*) in ABA, 0.6667 (WT vs. *OSA1#2*) in ABA, and 0.3919 (WT vs. *OSA1#3*) in ABA for **b**; 0.0991 (WT vs. *OSA1#1*) in DK, 0.2131 (WT vs. *OSA1#2*) in DK, 0.3548 (WT vs. *OSA1#3*) in DK, 0.0017 (WT vs. *OSA1#1*) in WL, 0.0031 (WT vs. *OSA1#2*) in WL, 0.0035 (WT vs. *OSA1#3*) in WL, 0.2490 (WT vs. *OSA1#1*) in 2nd DK, 0.3335 (WT vs. *OSA1#2*) in 2nd DK, and 0.3726 (WT vs. *OSA1#3*) in 2nd DK for **c**; 0.8458 (WT vs. *OSA1#1*) in DK, 0.9593 (WT vs. *OSA1#2*) in DK, 0.0816 (WT vs. *OSA1#3*) in DK, 0.0229 (WT vs. *OSA1#1*) in WL, 0.0550 (WT vs. *OSA1#2*) in WL, 0.0183 (WT vs. *OSA1#3*) in WL, 0.2769 (WT vs. *OSA1#1*) in 2nd DK, 0.3206 (WT vs. *OSA1#2*) in 2nd DK, and 0.7539 (WT vs. *OSA1#3*) in 2nd DK for **d**; 0.6122 (WT vs. *OSA1#1*) in 0 μmol m^-2^ s^-1^, 0.2759 (WT vs. *OSA1#2*) in 0 μmol m^-2^ s^-1^, 0.0270 (WT vs. *OSA1#3*) in 0 μmol m^-2^ s^-1^, 0.9924 (WT vs. *OSA1#1*) in 50 μmol m^-2^ s^-1^, 0.0970 (WT vs. *OSA1#2*) in 50 μmol m^-2^ s^-1^, 0.0701 (WT vs. *OSA1#3*) in 50 μmol m^-2^ s^-1^, 0.0629 (WT vs. *OSA1#1*) in 100 μmol m^-2^ s^-1^, 0.1345 (WT vs. *OSA1#2*) in 100 μmol m^-2^ s^-1^, 0.0752 (WT vs. *OSA1#3*) in 100 μmol m^-2^ s^-1^, 0.0345 (WT vs. *OSA1#1*) in 150 μmol m^-2^ s^-1^, 0.2019 (WT vs. *OSA1#2*) in 150 μmol m^-2^ s^-1^, 0.0592 (WT vs. *OSA1#3*) in 150 μmol m^-2^ s^-1^, 0.0258 (WT vs. *OSA1#1*) in 200 μmol m^-2^ s^-1^, 0.0881 (WT vs. *OSA1#2*) in 200 μmol m^-2^ s^-1^, 0.0590 (WT vs. *OSA1#3*) in 200 μmol m^-2^ s^-1^, 0.0090 (WT vs. *OSA1#1*) in 500 μmol m^-2^ s^-1^, 0.0427 (WT vs. *OSA1#2*) in 500 μmol m^-2^ s^-1^, 0.1980 (WT vs. *OSA1#3*) in 500 μmol m^-2^ s^-1^, 0.0796 (WT vs. *OSA1#1*) in 1 000 μmol m^-2^ s^-1^, 0.0166 (WT vs. *OSA1#2*) in 1 000 μmol m^-2^ s^-1^, 0.0403 (WT vs. *OSA1#3*) in 1 000 μmol m^-2^ s^-1^, 0.0890 (WT vs. *OSA1#1*) in 1 500 μmol m^-2^ s^-1^, 0.0271 (WT vs. *OSA1#2*) in 1 500 μmol m^-2^ s^-1^, and 0.0216 (WT vs. *OSA1#3*) in 1 500 μmol m^-2^ s^-1^ for **e**; 0.9380 (WT vs. *OSA1#1*) in 0 μmol m^-2^ s^-1^, 0.6034 (WT vs. *OSA1#2*) in 0 μmol m^-2^ s^-1^, 0.4668 (WT vs. *OSA1#3*) in 0 μmol m^-2^ s^-1^, 0.0739 (WT vs. *OSA1#1*) in 50 μmol m^-2^ s^-1^, 0.0498 (WT vs. *OSA1#2*) in 50 μmol m^-2^ s^-1^, 0.0061 (WT vs. *OSA1#3*) in 50 μmol m^-2^ s^-1^, 0.0144 (WT vs. *OSA1#1*) in 100 μmol m^-2^ s^-1^, 0.0439 (WT vs. *OSA1#2*) in 100 μmol m^-2^ s^-1^, 0.0486 (WT vs. *OSA1#3*) in 100 μmol m^-2^ s^-1^, 0.1319 (WT vs. *OSA1#1*) in 150 μmol m^-2^ s^-1^, 0.1669 (WT vs. *OSA1#2*) in 150 μmol m^-2^ s^-1^, 0.2983 (WT vs. *OSA1#3*) in 150 μmol m^-2^ s^-1^, 0.0622 (WT vs. *OSA1#1*) in 200 μmol m^-2^ s^-1^, 0.0577 (WT vs. *OSA1#2*) in 200 μmol m^-2^ s^-1^, 0.2327 (WT vs. *OSA1#3*) in 200 μmol m^-2^ s^-1^, 0.0473 (WT vs. *OSA1#1*) in 500 μmol m^-2^ s^-1^, 0.0083 (WT vs. *OSA1#2*) in 500 μmol m^-2^ s^-1^, 0.0289 (WT vs. *OSA1#3*) in 500 μmol m^-2^ s^-1^, 0.0096 (WT vs. *OSA1#1*) in 1 000 μmol m^-2^ s^-1^, 0.0093 (WT vs. *OSA1#2*) in 1 000 μmol m^-2^ s^-1^, 0.0169 (WT vs. *OSA1#3*) in 1 000 μmol m^-2^ s^-1^, 0.0198 (WT vs. *OSA1#1*) in 1 500 μmol m^-2^ s^-1^, 0.0117 (WT vs. *OSA1#2*) in 1 500 μmol m^-2^ s^-1^, and 0.0105 (WT vs. *OSA1#3*) in 1 500 μmol m^-2^ s^-1^ for **f**;

**4.**The exact *p* values in **FIG.5** are 1.2×10^−4^ (WT vs. *OSA1#1*) in roots, 0.0026 (WT vs. *OSA1#2*) in roots, 3.5×10^−4^ (WT vs. *OSA1#3*) in roots, 0.0013 (WT vs. *OSA1#1*) in leaves, 0.0123 (WT vs. *OSA1#2*) in leaves, and 0.0018 (WT vs. *OSA1#3*) in leaves for **c**; 0.0011 (WT vs. *OSA1#1*), 8.2×10^−5^ (WT vs. *OSA1#2*), and 3.0×10^−5^ (WT vs. *OSA1#3*) for **d**; 0.0018 (WT vs. *OSA1#1*), 6.8×10^−4^ (WT vs. *OSA1#2*), and 3.1×10^−3^ (WT vs. *OSA1#3*) for **e**; 0.0216 (WT vs. *OSA1#1*), 2.6×10^−4^ (WT vs. *OSA1#2*), and 5.2×10^−4^ (WT vs. *OSA1#3*) for **f**; 0.0012 (WT vs. *OSA1#1*), 4.6×10^−4^ (WT vs. *OSA1#2*), and 3.6×10^−4^ (WT vs. *OSA1#3*) for **g**; 8.1×10^−5^ (WT vs. *OSA1#1*), 5.5×10^−4^ (WT vs. *OSA1#2*), and 4.7×10^−4^ (WT vs. *OSA1#3*) for **h**.

**5.**The exact *p* values in **Fig.6** are 2.5×10^−4^ (WT vs. *OSA1#1*) in 2016 Nanjing-S, 1.2×10^−4^ (WT vs. *OSA1#2*) in 2016 Nanjing-S, 5.3×10^−5^ (WT vs. *OSA1#3*) in 2016 Nanjing-S, 0.0114 (WT vs. *OSA1#1*) in 2017 Nanjing-N, 0.0148 (WT vs. *OSA1#2*) in 2017 Nanjing-N, 0.0020 (WT vs. *OSA1#3*) in 2017 Nanjing-N, 1.5×10^−4^ (WT vs. *OSA1#1*) in 2017 Fengyang, 1.4×10^−6^ (WT vs. *OSA1#2*) in 2017 Fengyang, and 3.6×10^−7^ (WT vs. *OSA1#3*) in 2017 Fengyang for **e**; 6.4×10^−5^ (WT vs. *OSA1#1*) in 2016 Nanjing-S, 1.8×10^−6^ (WT vs. *OSA1#2*) in 2016 Nanjing-S, 1.2×10^−6^ (WT vs. *OSA1#3*) in 2016 Nanjing-S, 0.0055 (WT vs. *OSA1#1*) in 2017 Nanjing-N, 0.0092 (WT vs. *OSA1#2*) in 2017 Nanjing-N, 0.0063 (WT vs. *OSA1#3*) in 2017 Nanjing-N, 0.0020 (WT vs. *OSA1#1*) in 2017 Fengyang, 0.0016 (WT vs. *OSA1#2*) in 2017 Fengyang, and 1.8×10^−4^ (WT vs. *OSA1#3*) in 2017 Fengyang for **f**; 0.0122 (WT vs. *OSA1#1*) in 2016 Nanjing-S, 0.0170 (WT vs. *OSA1#2*) in 2016 Nanjing-S, 0.0165 (WT vs. *OSA1#3*) in 2016 Nanjing-S, 0.0390 (WT vs. *OSA1#1*) in 2017 Nanjing-N, 0.0525 (WT vs. *OSA1#2*) in 2017 Nanjing-N, 0.0247 (WT vs. *OSA1#3*) in 2017 Nanjing-N, 0.0082 (WT vs. *OSA1#1*) in 2017 Fengyang, 0.0001 (WT vs. *OSA1#2*) in 2017 Fengyang, and 0.0012 (WT vs. *OSA1#3*) in 2017 Fengyang for **g**; 0.0019 (WT vs. *OSA1#1*) in 2016 Nanjing-S, 5.2×10^−4^ (WT vs. *OSA1#2*) in 2016 Nanjing-S, 0.0054 (WT vs. *OSA1#3*) in 2016 Nanjing-S, 0.0066 (WT vs. *OSA1#1*) in 2017 Nanjing-N, 0.0017 (WT vs. *OSA1#2*) in 2017 Nanjing-N, 9.1×10^−4^ (WT vs. *OSA1#3*) in 2017 Nanjing-N, 5.5×10^−5^ (WT vs. *OSA1#1*) in 2017 Fengyang, 2.1×10^−5^ (WT vs. *OSA1#2*) in 2017 Fengyang, and 1.6×10^−5^ (WT vs. *OSA1#3*) in 2017 Fengyang for **h**; 0.0336 (WT vs. *OSA1#1*) in L-N 2016 Nanjing-S, 0.1307 (WT vs. *OSA1#2*) in L-N 2016 Nanjing-S, 0.0073 (WT vs. *OSA1#3*) in L-N 2016 Nanjing-S, 0.0148 (WT vs. *OSA1#1*) in M-N 2016 Nanjing-S, 0.0028 (WT vs. *OSA1#2*) in M-N 2016 Nanjing-S, 0.0019 (WT vs. *OSA1#3*) in M-N 2016 Nanjing-S, 0.0400 (WT vs. *OSA1#1*) in H-N 2016 Nanjing-S, 7.7×10 ^−4^ (WT vs. *OSA1#2*) in H-N 2016 Nanjing-S, 0.0084 (WT vs. *OSA1#3*) in H-N 2016 Nanjing-S,0.0210 (WT vs. *OSA1#1*) in L-N 2017 Nanjing-N, 0.0274 (WT vs. *OSA1#2*) in L-N 2017 Nanjing-N, 0.0653 (WT vs. *OSA1#3*) in L-N 2017 Nanjing-N, 0.0482 (WT vs. *OSA1#1*) in M-N 2017 Nanjing-N, 0.0034 (WT vs. *OSA1#2*) in M-N 2017 Nanjing-N, 0.0261(WT vs. *OSA1#3*) in M-N 2017 Nanjing-N, 0.0037 (WT vs. *OSA1#1*) in H-N 2017 Nanjing-N, 0.0047 (WT vs. *OSA1#2*) in H-N 2017 Nanjing-N, 0.0026 (WT vs. *OSA1#3*) in H-N 2017 Nanjing-N, 0.0391 (WT vs. *OSA1#1*) in L-N 2017 Fengyang, 0.0754 (WT vs. *OSA1#2*) in L-N 2017 Fengyang, 0.0825 (WT vs. *OSA1#3*) in L-N 2017 Fengyang, 0.0021 (WT vs. *OSA1#1*) in M-N 2017 Fengyang, 0.0066 (WT vs. *OSA1#2*) in M-N 2017 Fengyang, 0.0329 (WT vs. *OSA1#3*) in M-N 2017 Fengyang, 0.1044 (WT vs. *OSA1#1*) in H-N 2017 Fengyang, 0.0004 (WT vs. *OSA1#2*) in H-N 2017 Fengyang, and 0.0076 (WT vs. *OSA1#3*) in H-N 2017 Fengyang, for **i**; 0.0160 (WT vs. *OSA1-oxs*) in N-N 2016 Nanjing-S, 5.7×10^−6^ (WT vs. *OSA1-oxs*) in L-N 2016 Nanjing-S, 4.0×10^−8^ (WT vs. *OSA1-oxs*) in M-N 2016 Nanjing-S, 7.4×10^−4^ (WT vs. *OSA1-oxs*) in H-N 2016 Nanjing-S, 0.0011 (WT M-N vs. *OSA1-oxs* L-N) in 2016 Nanjing-S, 0.1220 (WT H-N vs. *OSA1-oxs* L-N) in 2016 Nanjing-S, 0.0020 (WT vs. *OSA1-oxs*) in N-N 2017 Nanjing-N, 2.4×10^−4^ (WT vs. *OSA1-oxs*) in L-N 2017 Nanjing-N, 5.0×10^−4^ (WT vs. *OSA1-oxs*) in M-N 2017 Nanjing-N, 1.6×10^−6^ (WT vs. *OSA1-oxs*) in H-N 2017 Nanjing-N, 0.0657 (WT M-N vs. *OSA1-oxs* L-N) in 2017 Nanjing-N, 0.9551 (WT H-N vs. *OSA1-oxs* L-N) in 2017 Nanjing-N,6.6×10^−4^ (WT vs. *OSA1-oxs*) in N-N 2017 Fengyang, 7.6×10^−6^ (WT vs. *OSA1-oxs*) in L-N 2017 Fengyang, 9.3×10^−9^ (WT vs. *OSA1-oxs*) in M-N 2017 Fengyang, 2.1×10^−7^ (WT vs. *OSA1-oxs*) in H-N 2017 Fengyang, 7.3×10^−4^ (WT M-N vs. *OSA1-oxs* L-N) in 2017 Fengyang, and 0.0729 (WT H-N vs. *OSA1-oxs* L-N) in 2017 Fengyang for **j**.

**6.**The exact *p* values in **supplementary figures** **2** are 5.5×10^−7^ (WT vs. *OSA1#1*), 3.4×10^−5^ (WT vs. *OSA1#2*), and 3.0×10^−6^ (WT vs. *OSA1#3*) for **b**; 0.0010 (WT vs. *OSA1#1*), 4.5×10^−5^ (WT vs. *OSA1#2*), 2.6×10^−4^ (WT vs. *OSA1#3*) in 1.53 min, 2.0×10^−3^ (WT vs. *OSA1#1*), 7.5×10^−4^ (WT vs. *OSA1#2*), 3.9×10^−4^ (WT vs. *OSA1#3*) in 1.60 min, 7.2×10^−4^ (WT vs. *OSA1#1*), 2.1×10^−4^ (WT vs. *OSA1#2*), 1.3×10^−4^ (WT vs. *OSA1#3*) in 1.67 min, 0.0026 (WT vs. *OSA1#1*), 5.7×10^−4^ (WT vs. *OSA1#2*), 0.0016 (WT vs. *OSA1#3*) in 1.73 min, 0164 (WT vs. *OSA1#1*), 2.1×10^−4^ (WT vs. *OSA1#2*), 4.7×10^−4^ (WT vs. *OSA1#3*) in 1.80 min, 3.5×10^−3^ (WT vs. *OSA1#1*), 4.3×10^−4^ (WT vs. *OSA1#2*), 0.0016 (WT vs. *OSA1#3*) in 1.87 min, 0.0258 (WT vs. *OSA1#1*), 0.0041 (WT vs. *OSA1#2*), 0.0061 (WT vs. *OSA1#3*) in 1.93 min, 0.0095 (WT vs. *OSA1#1*), 0.0041 (WT vs. *OSA1#2*), 1.5×10^−4^ (WT vs. *OSA1#3*) in 2.00 min, 0.0151 (WT vs. *OSA1#1*), 0.0390 (WT vs. *OSA1#2*), 0.0046 (WT vs. *OSA1#3*) in 2.07 min, 0.0046 (WT vs. *OSA1#1*), 0.0022 (WT vs. *OSA1#2*), 0.0043 (WT vs. *OSA1#3*) in 2.13 min, 0.0103 (WT vs. *OSA1#1*), 0.0092 (WT vs. *OSA1#2*), 0.0237 (WT vs. *OSA1#3*) in 2.20 min, 0.0022 (WT vs. *OSA1#1*), 0.0012 (WT vs. *OSA1#2*), 0.0052 (WT vs. *OSA1#3*) in 2.27 min, 0.0025 (WT vs. *OSA1#1*), 0.0100 (WT vs. *OSA1#2*), 0.0043 (WT vs. *OSA1#3*) in 2.33 min, 0.0028 (WT vs. *OSA1#1*), 0.0126 (WT vs. *OSA1#2*), 0.0012 (WT vs. *OSA1#3*) in 2.40 min, 0.0010 (WT vs. *OSA1#1*), 0.0134 (WT vs. *OSA1#2*), 1.7×10^−4^ (WT vs. *OSA1#3*) in 2.47 min, 0.0047 (WT vs. *OSA1#1*), 0.0046 (WT vs. *OSA1#2*), 0.0017 (WT vs. *OSA1#3*) in 2.53 min, 0.0028 (WT vs. *OSA1#1*), 0.0004 (WT vs. *OSA1#2*), 0.0004 (WT vs. *OSA1#3*) in 2.60 min, 0.0235 (WT vs. *OSA1#1*), 0.0596 (WT vs. *OSA1#2*), 0.0081 (WT vs. *OSA1#3*) in 2.67 min, 0.0059 (WT vs. *OSA1#1*), 0.0409 (WT vs. *OSA1#2*), 0.0004 (WT vs. *OSA1#3*) in 2.73 min, 0.0360 (WT vs. *OSA1#1*), 0.0290 (WT vs. *OSA1#2*), 0.0025 (WT vs. *OSA1#3*) in 2.80 min, 0.0022 (WT vs. *OSA1#1*), 0.0060 (WT vs. *OSA1#2*), 0.0002 (WT vs. *OSA1#3*) in 2.87 min, 0.0057 (WT vs. *OSA1#1*), 0.0031 (WT vs. *OSA1#2*), 0.0112 (WT vs. *OSA1#3*) in 2.93 min, 0.0176 (WT vs. *OSA1#1*), 0.0361 (WT vs. *OSA1#2*), 0.0029 (WT vs. *OSA1#3*) in 3.00 min, 0.0082 (WT vs. *OSA1#1*), 0.0424 (WT vs. *OSA1#2*), 0.0109 (WT vs. *OSA1#3*) in 3.07 min, 0.0096 (WT vs. *OSA1#1*), 0.1246 (WT vs. *OSA1#2*), 0.0091 (WT vs. *OSA1#3*) in 3.13 min, 0.0257 (WT vs. *OSA1#1*), 0.0325 (WT vs. *OSA1#2*), 0.0048 (WT vs. *OSA1#3*) in 3.20 min, 0.0139 (WT vs. *OSA1#1*), 0.2060 (WT vs. *OSA1#2*), 0.0060 (WT vs. *OSA1#3*) in 3.27 min, 0.0078 (WT vs. *OSA1#1*), 0.0340 (WT vs. *OSA1#2*), 0.0025 (WT vs. *OSA1#3*) in 3.33 min, 0.0004 (WT vs. *OSA1#1*), 0.0033 (WT vs. *OSA1#2*), 5.7×10^−4^ (WT vs. *OSA1#3*) in 3.40 min, 0.0012 (WT vs. *OSA1#1*), 0.0011 (WT vs. *OSA1#2*), 1.4×10^−4^ (WT vs. *OSA1#3*) in 3.47 min, 0.0019 (WT vs. *OSA1#1*), 0.0087 (WT vs. *OSA1#2*), 0.0031 (WT vs. *OSA1#3*) in 3.53 min, 2.7×10^−5^ (WT vs. *OSA1#1*), 0.0001 (WT vs. *OSA1#2*), 1.4×10^−4^ (WT vs. *OSA1#3*) in 3.60 min, 0.0006 (WT vs. *OSA1#1*), 5.4×10^−4^ (WT vs. *OSA1#2*), 1.4×10^−4^ (WT vs. *OSA1#3*) in 3.67 min, 0.0001 (WT vs. *OSA1#1*), 0.0021 (WT vs. *OSA1#2*), 1.6×10^−4^ (WT vs. *OSA1#3*) in 3.73 min, 1.5×10^−4^ (WT vs. *OSA1#1*), 1.1×10^−4^ (WT vs. *OSA1#2*), 6.9×10^−5^ (WT vs. *OSA1#3*) in 3.80 min, 5.3×10^−4^ (WT vs. *OSA1#1*), 4.3×10^−4^ (WT vs. *OSA1#2*), 1.8×10^−4^ (WT vs. *OSA1#3*) in 3.87 min, 2.1×10^−4^ (WT vs. *OSA1#1*), 6.2×10^−4^ (WT vs. *OSA1#2*), 3.5×10^−4^ (WT vs. *OSA1#3*) in 3.93 min, 1.5×10^−4^ (WT vs. *OSA1#1*), 6.6×10^−5^ (WT vs. *OSA1#2*), 6.7×10^−5^ (WT vs. *OSA1#3*) in 4.00 min, 0.0015 (WT vs. *OSA1#1*), 0.0031 (WT vs. *OSA1#2*), 0.0011 (WT vs. *OSA1#3*) in 4.07 min, 2.2×10^−4^ (WT vs. *OSA1#1*), 5.0×10^−4^ (WT vs. *OSA1#2*), 6.3×10^−4^ (WT vs. *OSA1#3*) in 4.13min, 1.0×10^−4^ (WT vs. *OSA1#1*), 2.4×10^−5^ (WT vs. *OSA1#2*), 2.4×10^−4^ (WT vs. *OSA1#3*) in 4.20 min, 3.2×10^−5^ (WT vs. *OSA1#1*), 5.5×10^−5^ (WT vs. *OSA1#2*), 6.5×10^−4^ (WT vs. *OSA1#3*) in 4.27 min, 1.7×10^−4^ (WT vs. *OSA1#1*), 2.5×10^−4^ (WT vs. *OSA1#2*), 9.6×10^−4^ (WT vs. *OSA1#3*) in 4.33 min, 5.9×10^−4^ (WT vs. *OSA1#1*), 5.3×10^−4^ (WT vs. *OSA1#2*), 0.0016 (WT vs. *OSA1#3*) in 4.40 min, 6.4×10^−5^ (WT vs. *OSA1#1*), 2.6×10^−5^ (WT vs. *OSA1#2*), 5.8×10^−5^ (WT vs. *OSA1#3*) in 4.47 min, 3.1×10^−5^ (WT vs. *OSA1#1*), 2.5×10^−6^ (WT vs. *OSA1#2*), 8.7×10^−5^ (WT vs. *OSA1#3*) in 4.53 min, 0.0001 (WT vs. *OSA1#1*), 1.1×10^−4^ (WT vs. *OSA1#2*), 2.6×10^−4^ (WT vs. *OSA1#3*) in 4.60 min, 1.7×10^−4^ (WT vs. *OSA1#1*), 3.6×10^−5^ (WT vs. *OSA1#2*), 4.3×10^−5^ (WT vs. *OSA1#3*) in 4.67 min, 8.4×10^−5^ (WT vs. *OSA1#1*), 1.1×10^−6^ (WT vs. *OSA1#2*), 2.2×10^−4^ (WT vs. *OSA1#3*) in 4.73 min, 1.4×10^−4^ (WT vs. *OSA1#1*), 9.5×10^−6^ (WT vs. *OSA1#2*), 2.9×10^−5^ (WT vs. *OSA1#3*) in 4.80 min, 0.0016 (WT vs. *OSA1#1*), 4.4×10^−4^ (WT vs. *OSA1#2*), 3.6×10^−4^ (WT vs. *OSA1#3*) in 4.87 min, 0.0012 (WT vs. *OSA1#1*), 2.2×10^−4^ (WT vs. *OSA1#2*), 3.3×10^−4^ (WT vs. *OSA1#3*) in 4.93 min, 0.0022 (WT vs. *OSA1#1*), 0.0025 (WT vs. *OSA1#2*), 1.8×10^−4^ (WT vs. *OSA1#3*) in 5.00 min, 3.2×10^−4^ (WT vs. *OSA1#1*), 7.6×10^−4^ (WT vs. *OSA1#2*), 8.3×10^−5^ (WT vs. *OSA1#3*) in 5.07 min, 2.9×10^−5^ (WT vs. *OSA1#1*), 4.1×10^−4^ (WT vs. *OSA1#2*), 4.5×10^−5^ (WT vs. *OSA1#3*) in 5.13 min, 7.9×10^−4^ (WT vs. *OSA1#1*), 1.7×10^−4^ (WT vs. *OSA1#2*), 3.6×10^−4^ (WT vs. *OSA1#3*) in 5.20 min, 2.6×10^−4^ (WT vs. *OSA1#1*), 0.0021 (WT vs. *OSA1#2*), 4.4×10^−4^ (WT vs. *OSA1#3*) in 5.27 min, 3.5×10^−4^ (WT vs. *OSA1#1*), 3.8×10^−4^ (WT vs. *OSA1#2*), 3.0×10^−5^ (WT vs. *OSA1#3*) in 5.33 min, 1.6×10^−5^ (WT vs. *OSA1#1*), 4.8×10^−6^ (WT vs. *OSA1#2*), 1.7×10^−6^ (WT vs. *OSA1#3*) in 5.40 min, 0.0013 (WT vs. *OSA1#1*), 3.9×10^−4^ (WT vs. *OSA1#2*), 1.2×10^−4^ (WT vs. *OSA1#3*) in 5.47 min, 2.0×10^−4^ (WT vs. *OSA1#1*), 1.8×10^−4^ (WT vs. *OSA1#2*), 2.5×10^−6^ (WT vs. *OSA1#3*) in 5.53 min, 9.9×10^−5^ (WT vs. *OSA1#1*), 2.9×10^−4^ (WT vs. *OSA1#2*), 2.7×10^−5^ (WT vs. *OSA1#3*) in 5.60 min, 1.8×10^−5^ (WT vs. *OSA1#1*), 0.0033 (WT vs. *OSA1#2*), 0.0092 (WT vs. *OSA1#3*) in 5.67 min, 0.0034 (WT vs. *OSA1#1*), 0.0012 (WT vs. *OSA1#2*), 7.1×10^−4^ (WT vs. *OSA1#3*) in 5.73 min, 3.8×10^−4^ (WT vs. *OSA1#1*), 4.3×10^−4^ (WT vs. *OSA1#2*), 8.0×10^−5^ (WT vs. *OSA1#3*) in 5.80 min, 0.0027 (WT vs. *OSA1#1*), 1.2×10^−6^ (WT vs. *OSA1#2*), 3.3×10^−5^ (WT vs. *OSA1#3*) in 5.87 min, 0.0020 (WT vs. *OSA1#1*), 4.1×10^−4^ (WT vs. *OSA1#2*), 6.3×10^−4^ (WT vs. *OSA1#3*) in 5.93 min, 2.3×10^−4^ (WT vs. *OSA1#1*), 5.9×10^−7^ (WT vs. *OSA1#2*), 5.9×10^−6^ (WT vs. *OSA1#3*) in 6.00 min, 0.0010 (WT vs. *OSA1#1*), 4.8×10^−6^ (WT vs. *OSA1#2*), 1.8×10^−6^ (WT vs. *OSA1#3*) in 6.07 min, 6.2×10^−5^ (WT vs. *OSA1#1*), 1.6×10^−4^ (WT vs. *OSA1#2*), 3.8×10^−6^ (WT vs. *OSA1#3*) in 6.13 min, 0.0045 (WT vs. *OSA1#1*), 9.0×10^−4^ (WT vs. *OSA1#2*), 7.2×10^−4^ (WT vs. *OSA1#3*) in 6.20 min, 0.0004 (WT vs. *OSA1#1*), 1.1×10^−5^ (WT vs. *OSA1#2*), 5.3×10^−6^ (WT vs. *OSA1#3*) in 6.27 min, 0.0027 (WT vs. *OSA1#1*), 0.0014 (WT vs. *OSA1#2*), 9.3×10^−5^ (WT vs. *OSA1#3*) in 6.33 min, 0.0018 (WT vs. *OSA1#1*), 9.9×10^−5^ (WT vs. *OSA1#2*), 1.1×10^−5^ (WT vs. *OSA1#3*) in 6.40 min, 0.0043 (WT vs. *OSA1#1*), 0.0031 (WT vs. *OSA1#2*), 8.0×10^−6^ (WT vs. *OSA1#3*) in 6.47 min, 5.1×10^−4^ (WT vs. *OSA1#1*), 1.4×10^−4^ (WT vs. *OSA1#2*), 8.8×10^−6^ (WT vs. *OSA1#3*) in 6.53 min, 2.3×10^−4^ (WT vs. *OSA1#1*), 1.2×10^−4^ (WT vs. *OSA1#2*), 3.1×10^−5^ (WT vs. *OSA1#3*) in 6.60 min, 8.7×10^−5^ (WT vs. *OSA1#1*), 2.8×10^−4^ (WT vs. *OSA1#2*), 3.1×10^−5^ (WT vs. *OSA1#3*) in 6.67 min, 7.3×10^−4^ (WT vs. *OSA1#1*), 8.9×10^−4^ (WT vs. *OSA1#2*), 0.0034 (WT vs. *OSA1#3*) in 6.73 min, 0.0046 (WT vs. *OSA1#1*), 5.1×10^−4^ (WT vs. *OSA1#2*), 2.3×10^−4^ (WT vs. *OSA1#3*) in 6.80 min, 6.3×10^−4^ (WT vs. *OSA1#1*), 1.1×10^−5^ (WT vs. *OSA1#2*), 6.2×10^−5^ (WT vs. *OSA1#3*) in 6.87 min, 7.3×10^−4^ (WT vs. *OSA1#1*), 5.2×10^−4^ (WT vs. *OSA1#2*), 1.2×10^−5^ (WT vs. *OSA1#3*) in 6.93 min, 0.0012 (WT vs. *OSA1#1*), 2.1×10^−4^ (WT vs. *OSA1#2*), 6.3×10^−5^ (WT vs. *OSA1#3*) in 7.00 min, 3.4×10^−4^ (WT vs. *OSA1#1*), 5.6×10^−5^ (WT vs. *OSA1#2*), 5.5×10^−5^ (WT vs. *OSA1#3*) in 7.07 min, 0.0098 (WT vs. *OSA1#1*), 0.0074 (WT vs. *OSA1#2*), 8.6×10^−4^ (WT vs. *OSA1#3*) in 7.13 min, 0.0184 (WT vs. *OSA1#1*), 0.0107 (WT vs. *OSA1#2*), 0.0049 (WT vs. *OSA1#3*) in 7.20 min, 3.1×10^−4^ (WT vs. *OSA1#1*), 4.2×10^−5^ (WT vs. *OSA1#2*), 1.1×10^−5^ (WT vs. *OSA1#3*) in 7.27 min,0.0027 (WT vs. *OSA1#1*), 0.0012 (WT vs. *OSA1#2*), 0.0044 (WT vs. *OSA1#3*) in 7.33 min, 0.0021 (WT vs. *OSA1#1*), 1.6×10^−6^ (WT vs. *OSA1#2*), 1.7×10^−5^ (WT vs. *OSA1#3*) in 7.40 min, 1.0×10^−5^ (WT vs. *OSA1#1*), 9.3×10^−4^ (WT vs. *OSA1#2*), 4.1×10^−4^ (WT vs. *OSA1#3*) in 7.47 min, 7.0×10^−5^ (WT vs. *OSA1#1*), 7.7×10^−5^ (WT vs. *OSA1#2*), 2.9×10^−5^ (WT vs. *OSA1#3*) in 7.53 min, 1.1×10^−4^ (WT vs. *OSA1#1*), 0.0018 (WT vs. *OSA1#2*), 4.2×10^−5^ (WT vs. *OSA1#3*) in 7.60 min, 4.4×10^−4^ (WT vs. *OSA1#1*), 1.7×10^−4^ (WT vs. *OSA1#2*), 7.0×10^−5^ (WT vs. *OSA1#3*) in 7.67 min, 7.8×10^−5^ (WT vs. *OSA1#1*), 1.2×10^−4^ (WT vs. *OSA1#2*), 1.7×10^−6^ (WT vs. *OSA1#3*) in 7.73 min, 0.0070 (WT vs. *OSA1#1*), 0.0017 (WT vs. *OSA1#2*), 0.0033 (WT vs. *OSA1#3*) in 7.80 min, 0.0546 (WT vs. *OSA1#1*), 0.0086 (WT vs. *OSA1#2*), 0.0111 (WT vs. *OSA1#3*) in 7.87 min, 0.0011 (WT vs. *OSA1#1*), 2.7×10^−6^ (WT vs. *OSA1#2*), 1.2×10^−6^ (WT vs. *OSA1#3*) in 7.93 min, 3.8×10^−4^ (WT vs. *OSA1#1*), 0.0045 (WT vs. *OSA1#2*), 1.8×10^−5^ (WT vs. *OSA1#3*) in 8.00 min, 0.0135 (WT vs. *OSA1#1*), 0.0072 (WT vs. *OSA1#2*), 0.0100 (WT vs. *OSA1#3*) in 8.07 min, 0.0019 (WT vs. *OSA1#1*), 0.0140 (WT vs. *OSA1#2*), 5.0×10^−4^ (WT vs. *OSA1#3*) in 8.13 min, 1.1×10^−4^ (WT vs. *OSA1#1*), 5.1×10^−4^ (WT vs. *OSA1#2*), 3.2×10^−5^ (WT vs. *OSA1#3*) in 8.20 min, 0.0011 (WT vs. *OSA1#1*), 3.9×10^−5^ (WT vs. *OSA1#2*), 9.8×10^−5^ (WT vs. *OSA1#3*) in 8.27 min,0.0010 (WT vs. *OSA1#1*), 3.3×10^−5^ (WT vs. *OSA1#2*), 6.3×10^−4^ (WT vs. *OSA1#3*) in 8.33 min, 1.8×10^−5^ (WT vs. *OSA1#1*), 1.3×10^−4^ (WT vs. *OSA1#2*), 3.3×10^−4^ (WT vs. *OSA1#3*) in 8.40 min, 8.9×10^−5^ (WT vs. *OSA1#1*), 0.0015 (WT vs. *OSA1#2*), 0.001 (WT vs. *OSA1#3*) in 8.47 min, 0.0018 (WT vs. *OSA1#1*), 0.0116 (WT vs. *OSA1#2*), 1.5×10^−4^ (WT vs. *OSA1#3*) in 8.53 min, 7.2×10^−6^ (WT vs. *OSA1#1*), 4.8×10^−5^ (WT vs. *OSA1#2*), 9.0×10^−4^ (WT vs. *OSA1#3*) in 8.60 min, 7.1×10^−4^ (WT vs. *OSA1#1*), 1.2×10^−5^ (WT vs. *OSA1#2*), 4.2×10^−4^ (WT vs. *OSA1#3*) in 8.67 min, 0.0024 (WT vs. *OSA1#1*), 6.8×10^−6^ (WT vs. *OSA1#2*), 2.4×10^−5^ (WT vs. *OSA1#3*) in 8.73 min, 1.3×10^−4^ (WT vs. *OSA1#1*), 0.0011 (WT vs. *OSA1#2*), 1.6×10^−4^ (WT vs. *OSA1#3*) in 8.80 min, 0.0019 (WT vs. *OSA1#1*), 3.8×10^−5^ (WT vs. *OSA1#2*), 1.6×10^−5^ (WT vs. *OSA1#3*) in 8.87 min, 2.2×10^−5^ (WT vs. *OSA1#1*), 2.1×10^−6^ (WT vs. *OSA1#2*), 7.1×10^−5^ (WT vs. *OSA1#3*) in 8.93 min, 5.6×10^−5^ (WT vs. *OSA1#1*), 3.6×10^−4^ (WT vs. *OSA1#2*), 7.3×10^−4^ (WT vs. *OSA1#3*) in 9.00 min, 1.8×10^−4^ (WT vs. *OSA1#1*), 1.1×10^−4^ (WT vs. *OSA1#2*), 1.6×10^−4^ (WT vs. *OSA1#3*) in 9.07 min, 3.2×10^−4^ (WT vs. *OSA1#1*), 9.1×10^−4^ (WT vs. *OSA1#2*), 0.0018 (WT vs. *OSA1#3*) in 9.13 min, 0.0069 (WT vs. *OSA1#1*), 6.5×10^−4^ (WT vs. *OSA1#2*), 6.1×10^−4^ (WT vs. *OSA1#3*) in 9.20 min, 0.0012 (WT vs. *OSA1#1*), 2.5×10^−6^ (WT vs. *OSA1#2*), 9.2×10^−5^ (WT vs. *OSA1#3*) in 9.27 min,0.0013 (WT vs. *OSA1#1*), 9.3×10^−4^ (WT vs. *OSA1#2*), 8.4×10^−4^ (WT vs. *OSA1#3*) in 9.33 min, 9.6×10^−5^ (WT vs. *OSA1#1*), 1.9×10^−4^ (WT vs. *OSA1#2*), 6.7×10^−5^ (WT vs. *OSA1#3*) in 9.40 min, 2.5×10^−5^ (WT vs. *OSA1#1*), 4.5×10^−6^ (WT vs. *OSA1#2*), 9.8×10^−5^ (WT vs. *OSA1#3*) in 9.47 min, 4.0×10^−5^ (WT vs. *OSA1#1*), 3.7×10^−4^ (WT vs. *OSA1#2*), 7.3×10^−5^ (WT vs. *OSA1#3*) in 9.53 min, 3.0×10^−4^ (WT vs. *OSA1#1*), 1.0×10^−4^ (WT vs. *OSA1#2*), 1.5×10^−5^ (WT vs. *OSA1#3*) in 9.60 min, 0.0022 (WT vs. *OSA1#1*), 3.4×10^−4^ (WT vs. *OSA1#2*), 1.9×10^−4^ (WT vs. *OSA1#3*) in 9.67 min, 0.0069 (WT vs. *OSA1#1*), 8.4×10^−4^ (WT vs. *OSA1#3*) in 9.73 min, 0.0378 (WT vs. *OSA1#1*), 1.5×10^−4^ (WT vs. *OSA1#3*) in 9.80 min, and 5.2×10^−4^ (WT vs. *OSA1#3*) in 9.87 min for **g.**

**7.**The exact *p* values in **Supplementary Figure 5** are 0.0140 (WT vs. *OSA1#1*), 0.0047 (WT vs. *OSA1#2*), 0.0118 (WT vs. *OSA1#3*), 0.0038 (WT vs. *osa1-1*), 0.0109 (WT vs. *osa1-2*), 0.0215 (WT vs. *osa1-3*) in Mock, 0.7223 (WT vs. *OSA1#1*), 0.9784 (WT vs. *OSA1#2*), 0.7450 (WT vs. *OSA1#3*), 0.0545 (WT vs. *osa1-1*), 0.2067 (WT vs. *osa1-2*), and 0.5970 (WT vs. *osa1-3*) in VA.

**8.**The exact *p* values in **Supplementary Figure 6** are 0.0141 (WT vs. *OSA1#1*), 0.0033 (WT vs. *OSA1#2*), 0.0092 (WT vs. *OSA1#3*), 0.0017 (WT vs. *osa1-1*), 0.0007 (WT vs. *osa1-2*), 0.0013 (WT vs. *osa1-3*) in roots, 0.0035 (WT vs. *OSA1#1*), 0.0108 (WT vs. *OSA1#2*), 0.0397 (WT vs. *OSA1#3*), 0.0002 (WT vs. *osa1-1*), 0.0020 (WT vs. *osa1-2*), and 0.0002 (WT vs. *osa1-3*) in leaves for **a**; 0.0017 (WT vs. *OSA1#1*), 0.0003 (WT vs. *OSA1#2*), 0.0022 (WT vs. *OSA1#3*), 0.0052 (WT vs. *osa1-1*), 0.0002 (WT vs. *osa1-2*), 0.0008 (WT vs. *osa1-3*) in roots, 0.0074 (WT vs. *OSA1#1*), 0.0434 (WT vs. *OSA1#2*), 0.0107 (WT vs. *OSA1#3*), 0.0027 (WT vs. *osa1-1*), 0.0331 (WT vs. *osa1-2*), and 0.0129 (WT vs. *osa1-3*) in leaves for **b**; 0.1190 (WT vs. *OSA1#1*), 0.0200 (WT vs. *OSA1#2*), 0.0434 (WT vs. *OSA1#3*), 0.0086 (WT vs. *osa1-1*), 0.0034 (WT vs. *osa1-2*), 0.0023 (WT vs. *osa1-3*) in roots, 0.0103 (WT vs. *OSA1#1*), 0.0114 (WT vs. *OSA1#2*), 0.0097 (WT vs. *OSA1#3*), 0.0035 (WT vs. *osa1-1*), 0.0035 (WT vs. *osa1-2*), and 0.0034 (WT vs. *osa1-3*) in leaves for **c**; 0.0740 (WT vs. *OSA1#1*), 0.0255 (WT vs. *OSA1#2*), 0.0300 (WT vs. *OSA1#3*), 0.0593 (WT vs. *osa1-1*), 0.0073 (WT vs. *osa1-2*), 0.0196 (WT vs. *osa1-3*) in roots, 0.0916 (WT vs. *OSA1#1*), 0.2145 (WT vs. *OSA1#2*), 0.1196 (WT vs. *OSA1#3*), 0.0022 (WT vs. *osa1-1*), 0.0288 (WT vs. *osa1-2*), and 0.0116 (WT vs. *osa1-3*) in leaves for **d**; 0.3070 (WT vs. *OSA1#1*), 0.0603 (WT vs. *OSA1#2*), 0.1331 (WT vs. *OSA1#3*), 0.0252 (WT vs. *osa1-1*), 0.0064 (WT vs. *osa1-2*), 0.0415 (WT vs. *osa1-3*) in roots, 0.0173 (WT vs. *OSA1#1*), 0.0297 (WT vs. *OSA1#2*), 0.1289 (WT vs. *OSA1#3*), 0.0039 (WT vs. *osa1-1*), 0.0310 (WT vs. *osa1-2*), and 0.0136 (WT vs. *osa1-3*) in leaves for **e**; 0.0427 (WT vs. *OSA1#1*), 0.0145 (WT vs. *OSA1#2*), 0.0276 (WT vs. *OSA1#3*), 0.0010 (WT vs. *osa1-1*), 0.0004 (WT vs. *osa1-2*), 0.0009 (WT vs. *osa1-3*) in roots, 0.0841 (WT vs. *OSA1#1*), 0.0340 (WT vs. *OSA1#2*), 0.1351 (WT vs. *OSA1#3*), 0.0013 (WT vs. *osa1-1*), 0.0431 (WT vs. *osa1-2*), and 0.0061 (WT vs. *osa1-3*) in leaves for **f**; 0.0192 (WT vs. *OSA1#1*), 0.0052 (WT vs. *OSA1#2*), 0.0054 (WT vs. *OSA1#3*), 0.0010 (WT vs. *osa1-1*), 0.0014 (WT vs. *osa1-2*), 0.0042 (WT vs. *osa1-3*) in roots, 0.1732 (WT vs. *OSA1#1*), 0.8946 (WT vs. *OSA1#2*), 0.0865 (WT vs. *OSA1#3*), 0.0143 (WT vs. *osa1-1*), 0.1250 (WT vs. *osa1-2*), and 0.0665 (WT vs. *osa1-3*) in leaves for **g**; 0.2091 (WT vs. *OSA1#1*), 0.2327 (WT vs. *OSA1#2*), 0.0943 (WT vs. *OSA1#3*), 0.1489 (WT vs. *osa1-1*), 0.0465 (WT vs. *osa1-2*), 0.0273 (WT vs. *osa1-3*) in roots, 0.0754 (WT vs. *OSA1#1*), 0.1655 (WT vs. *OSA1#2*), 0.1030 (WT vs. *OSA1#3*), 0.0065 (WT vs. *osa1-1*), 0.0193 (WT vs. *osa1-2*), and 0.0155 (WT vs. *osa1-3*) in leaves for **h**; 0.5908 (WT vs. *OSA1#1*), 0.3271 (WT vs. *OSA1#2*), 0.9269 (WT vs. *OSA1#3*), 0.0113 (WT vs. *osa1-1*), 0.0099 (WT vs. *osa1-2*), 0.0088 (WT vs. *osa1-3*) in roots, 0.7721 (WT vs. *OSA1#1*), 0.2391 (WT vs. *OSA1#2*), 0.0482 (WT vs. *OSA1#3*), 0.1918 (WT vs. *osa1-1*), 0.3851 (WT vs. *osa1-2*), and 0.0353 (WT vs. *osa1-3*) in leaves for **i**; 0.0837 (WT vs. *OSA1#1*), 0.0326 (WT vs. *OSA1#2*), 0.0094 (WT vs. *OSA1#3*), 0.0873 (WT vs. *osa1-1*), 0.0126 (WT vs. *osa1-2*), 0.0176 (WT vs. *osa1-3*) in roots, 0.0058 (WT vs. *OSA1#1*), 0.0874 (WT vs. *OSA1#2*), 0.0041 (WT vs. *OSA1#3*), 0.0018 (WT vs. *osa1-1*), 0.0512 (WT vs. *osa1-2*), and 0.0118 (WT vs. *osa1-3*) in leaves for **j**; 0.2875 (WT vs. *OSA1#1*), 0.0706 (WT vs. *OSA1#2*), 0.2093 (WT vs. *OSA1#3*), 0.0230 (WT vs. *osa1-1*), 0.0393 (WT vs. *osa1-2*), 0.0041 (WT vs. *osa1-3*) in roots, 0.8176 (WT vs. *OSA1#1*), 0.2678 (WT vs. *OSA1#2*), 0.2153 (WT vs. *OSA1#3*), 0.0096 (WT vs. *osa1-1*), 0.0270 (WT vs. *osa1-2*), and 0.0329 (WT vs. *osa1-3*) in leaves for **k**.

**9.**The exact *p* values in **Supplementary Figure 7** are 0.3108 (WT vs. *osa1-1*) in DK, 0.4867 (WT vs. *osa1-2*) in DK, 0.2431 (WT vs. *osa1-3*) in DK, 0.0038 (WT vs. *osa1-1*) in RL+BL, 0.0075 (WT vs. *osa1-2*) in RL+BL, 0.0025 (WT vs. *osa1-3*) in RL+BL, 0.0316 (WT vs. *osa1-1*) in ABA, 0.4943 (WT vs. *osa1-2*) in ABA, and 0.1211 (WT vs. *osa1-3*) in ABA for **a**; 0.5600 (WT vs. *osa1-1*) in DK, 0.0432 (WT vs. *osa1-2*) in DK, 0.3592 (WT vs. *osa1-3*) in DK, 0.0233 (WT vs. *osa1-1*) in WL, 0.0243 (WT vs. *osa1-2*) in WL, 0.1067 (WT vs. *osa1-3*) in WL, 0.9979 (WT vs. *osa1-1*) in 2nd DK, 0.1691 (WT vs. *osa1-2*) in 2nd DK, and 0.1513 (WT vs. *osa1-3*) in 2nd DK for **b**; 0.3737 (WT vs. *osa1-1*) in DK, 0.3179 (WT vs. *osa1-2*) in DK, 0.7542 (WT vs. *osa1-3*) in DK, 0.0013 (WT vs. *osa1-1*) in WL, 0.0029 (WT vs. *osa1-2*) in WL, 0.0025 (WT vs. *osa1-3*) in WL, 0.5949 (WT vs. *osa1-1*) in 2nd DK, 0.7468 (WT vs. *osa1-2*) in 2nd DK, and 0.7280 (WT vs. *osa1-3*) in 2nd DK for **c.**

**10.**The exact *p* values in **Supplementary Figure 10** are 0.1121 (WT vs. *OSA1#1*) in N-N, 0.0308 (WT vs. *OSA1#2*) in N-N, 0.0113 (WT vs. *OSA1#3*) in N-N, 0.8874 (WT vs. *osa1-1*) in N-N, 0.5931 (WT vs. *osa1-2*) in N-N, 0.2547 (WT vs. *osa1-3*) in N-N, 0.0029 (WT vs. *OSA1#1*) in L-N, 0.0021 (WT vs. *OSA1#2*) in L-N, 3.7×10^−5^ (WT vs. *OSA1#3*) in L-N, 0.0073 (WT vs. *osa1-1*) in L-N, 0.4262 (WT vs. *osa1-2*) in L-N, 0.0213 (WT vs. *osa1-3*) in L-N, 0.0608 (WT vs. *OSA1#1*) in M-N, 0.0060 (WT vs. *OSA1#2*) in M-N, 1.2×10^−4^ (WT vs. *OSA1#3*) in M-N, 0.0085 (WT vs. *osa1-1*) in M-N, 0.0497 (WT vs. *osa1-2*) in M-N, 0.0470 (WT vs. *osa1-3*) in M-N, 0.0101 (WT vs. *OSA1#1*) in H-N, 0.0109 (WT vs. *OSA1#2*) in H-N, 0.0077 (WT vs. *OSA1#3*) in H-N, 0.1398 (WT vs. *osa1-1*) in H-N, 0.0079 (WT vs. *osa1-2*) in H-N, and 0.0194 (WT vs. *osa1-3*) in H-N for **a**; 0.0858 (WT vs. *OSA1#1*) in N-N, 0.0789 (WT vs. *OSA1#2*) in N-N, 0.1322 (WT vs. *OSA1#3*) in N-N, 0.0394 (WT vs. *osa1-1*) in N-N, 0.3087 (WT vs. *osa1-2*) in N-N, 0.0200 (WT vs. *osa1-3*) in N-N, 0.0424 (WT vs. *OSA1#1*) in L-N, 0.0426 (WT vs. *OSA1#2*) in L-N, 0.0533 (WT vs. *OSA1#3*) in L-N, 0.0449 (WT vs. *osa1-1*) in L-N, 0.0531 (WT vs. *osa1-2*) in L-N, 0.4465 (WT vs. *osa1-3*) in L-N, 0.0108 (WT vs. *OSA1#1*) in M-N, 0.0124 (WT vs. *OSA1#2*) in M-N, 0.0097 (WT vs. *OSA1#3*) in M-N, 0.0317 (WT vs. *osa1-1*) in M-N, 0.0427 (WT vs. *osa1-2*) in M-N, 0.0168 (WT vs. *osa1-3*) in M-N, 0.0124 (WT vs. *OSA1#1*) in H-N, 0.0111 (WT vs. *OSA1#2*) in H-N, 0.0070 (WT vs. *OSA1#3*) in H-N, 0.0394 (WT vs. *osa1-1*) in H-N, 0.0066 (WT vs. *osa1-2*) in H-N, and 0.0330 (WT vs. *osa1-3*) in H-N for **b**.
